# Growth hormone-releasing hormone attenuates amyloid deposition and neuroinflammation in Alzheimer’s disease models

**DOI:** 10.1038/s41419-026-08699-w

**Published:** 2026-04-07

**Authors:** Francesca Pedrolli, Giulia Morello, Iacopo Gesmundo, Dana Banfi, Alma Ferro, Medhi Wangpaichitr, Wei Sha, Elena Tamagno, Andrew V. Schally, Michela Guglielmotto, Riccarda Granata

**Affiliations:** 1 Division of Endocrinology, Diabetes and Metabolism, Department of Medical Sciences, University of Turin, Turin, Italy; 2 Department of Neurosciences “Rita Levi Montalcini”, University of Turin and Neuroscience Institute Cavalieri Ottolenghi, Turin, Italy; 3 Miami VA Healthcare System, Endocrine and Polypeptide Institute, Miami, FL USA; 4 South Florida VA Foundation for Research and Education, Miami, FL USA; 5 Department of Medicine, Divisions of Medical/Oncology and Endocrinology, and the Department of Pathology, University of Miami, Miami, FL USA; 6 Sylvester Comprehensive Cancer Center, University of Miami, Miami, FL USA; 7 Department of Surgery, Division of Thoracic Surgery, University of Miami, Miami, FL USA; 8 Department of Molecular Biotechnology and Health Sciences, University of Turin, Turin, Italy

**Keywords:** Endocrinology, Experimental models of disease

## Abstract

Alzheimer’s disease (AD) is a progressive neurodegenerative disorder characterized by amyloid-β (Aβ) accumulation, tau hyperphosphorylation, neuroinflammation, and synaptic loss. Existing therapies provide only modest symptomatic relief and fail to slow disease progression. Beyond its role in promoting pituitary growth hormone (GH) secretion, growth hormone-releasing hormone (GHRH) has shown neuroprotective effects in experimental ischemic stroke and spinal muscular atrophy. Here, we explored the therapeutic potential of GHRH and its agonist MR-409 in AD models. In vitro, GHRH(1-44)NH₂ promoted survival, proliferation, and neuronal differentiation of rat hippocampal neural stem cells (NSCs) and human SH-SY5Y neuroblastoma cells under growth factor deprivation and amyloid beta (Aβ)_1-42_ exposure. These effects involved the cAMP/PKA/CREB, ERK1/2, and PI3K/Akt signaling pathways. GHRH also attenuated Aβ-induced neurotoxicity by reducing apoptosis, suppressing GSK-3β activity and tau phosphorylation, restoring nuclear β-catenin, and inhibiting NF-κB-mediated inflammation. In vivo, subcutaneous administration of MR-409 in 5xFAD mice reduced Aβ deposition, tau phosphorylation, gliosis, and proinflammatory cytokine expression. In addition, MR-409 mitigated neuronal and synaptic loss, activated survival and neurogenic pathways, and improved cognitive performance, without altering systemic GH and IGF1 levels. MR-409 also elevated NRF2 mRNA expression while reducing its negative regulator KEAP1. Overall, these findings indicate that GHRH and its analog MR-409 exert neuroprotective effects by modulating key pathological features of AD, including neurodegeneration, impaired neurogenesis, neuroinflammation, and oxidative stress. Given their ability to modulate multiple pathological pathways, GHRH agonists may represent promising therapeutic candidates for AD and other neurodegenerative disorders.

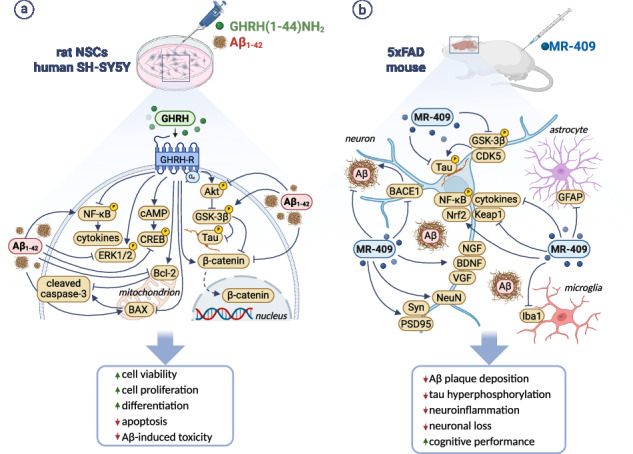

## Introduction

Alzheimer’s disease (AD) is a severe neurodegenerative disorder and the leading cause of dementia worldwide [1]. Its main pathological features are the accumulation of amyloid-β (Aβ) plaques and intraneuronal neurofibrillary tangles (NFTs) composed of hyperphosphorylated tau protein, which contribute to neuroinflammation, oxidative stress, and the degeneration of synapses and neurons, ultimately leading to cognitive decline [2, 3]. A rare familial early-onset form results from mutations in Aβ-processing genes amyloid precursor protein (*APP*), and presenilin 1 and 2 (*PSEN1* and *PSEN2*), while the more common late-onset form is influenced by aging, apolipoprotein E (APOE) ε4, and various environmental and metabolic factors [4, 5].

Multiple molecular pathways contribute to AD pathogenesis, including nuclear factor-κB (NF-κB) and glycogen synthase kinase-3 beta (GSK-3β). NF-κB, a central regulator of neuroinflammation, synaptic dysfunction, apoptosis, and oxidative stress, is activated by proinflammatory cytokines and Aβ plaques, leading to increased expression of β-site amyloid precursor protein cleaving enzyme-1 (BACE1) and enhanced amyloid production [6]. GSK-3β dysregulation promotes tau hyperphosphorylation and Aβ deposition [7], while its elevated activity in AD suppresses Wnt/β-catenin signaling, reducing neuroprotection and amplifying NF-κB-mediated inflammation [8].

Despite extensive research efforts, disease-modifying options for AD remain limited, and its incidence continues to increase with aging populations. Current therapies, including cholinesterase inhibitors, memantine, and anti-amyloid antibodies, provide modest symptomatic benefit, and while anti-amyloid antibodies have shown small but measurable effects on slowing progression in early AD, their overall clinical impact remains limited, and they may be associated with notable adverse effects [1, 5, 9]. Due to these limitations, research is shifting toward polytherapy or multi-target drugs to address different aspects of AD pathogenesis.

Growth hormone-releasing hormone (GHRH) is a hypothalamic neuropeptide that promotes the synthesis and release of growth hormone (GH) from the anterior pituitary [10]. GH, in turn, stimulates insulin-like growth factor 1 (IGF1) production from the liver. GHRH, GH, and IGF1 support brain development, neuronal growth, and neurotransmitter activity, playing key roles in central nervous system (CNS) function. Additionally, age-related declines in GH and IGF1 are linked to cognitive impairment and neurodegenerative diseases like AD [11–13].

GHRH and its receptors (GHRH-Rs), including the splice variant 1 (SV1), are found in various peripheral tissues, including the heart, muscle, pancreatic beta cells, and neurons, indicating extrapituitary functions [14]. In the brain, GHRH-R is present in areas such as the hypothalamus, hippocampus, cortex, and spinal cord, where it contributes to cognition, neuroprotection, and mood regulation [11, 15, 16]. GHRH also increases hypothalamic γ-aminobutyric acid (GABA) levels, activates GABA receptors, and promotes sleep [17, 18]. Furthermore, its presence in inhibitory neurons of epilepsy patients and mouse models of epilepsy, where it interacts with GABARs, suggests an antiepileptic role [19]. Interestingly, in APP mouse models of AD, GHRH improved sleep and reduced Aβ deposition [20]. Additionally, clinical studies have shown that GHRH analogs like Tesamorelin and GHRH(1–29)NH₂ enhance cognition in older adults and individuals with mild cognitive decline, suggesting therapeutic potential for neurodegenerative diseases [21, 22].

GHRH and its latest MR-class of agonists, developed by Dr. Andrew Schally’s laboratory, show strong receptor affinity and high stability, without activating the GH axis or inducing tumor growth [14, 23, 24]. These compounds promote cell growth and survival, while showing anti-inflammatory, cardioprotective, antidiabetic, and neuroprotective effects [11, 23–25]. MR-409, the most potent agonist, promotes neuroprotection in diabetic retinopathy models and shows anxiolytic and antidepressant effects [26–28]. It also enhances neural stem cell (NSC) proliferation, reduces mortality, and improves recovery in cerebral ischemia models [16]. Recently, we demonstrated that MR-409 alleviates spinal muscular atrophy (SMA) in mice by improving muscle trophism and neuromuscular junction alterations, preserving motor neurons, and attenuating inflammation [15]. Overall, these findings indicate that GHRH and MR-409 have neuroprotective potential, although their role in AD is still unknown.

This study investigated the role of GHRH in promoting neurogenesis and protecting rat hippocampal NSCs and human neuroblastoma cells from Aβ toxicity. Additionally, we evaluated whether MR-409 could mitigate key hallmarks of AD, including Aβ accumulation, tau hyperphosphorylation, neuroinflammation, and synaptic dysfunction in 5xFAD transgenic mice, and explored the underlying mechanisms.

## Results

### GHRH enhances NSC viability and differentiation through multiple signaling mechanisms

**Fig. 1:**
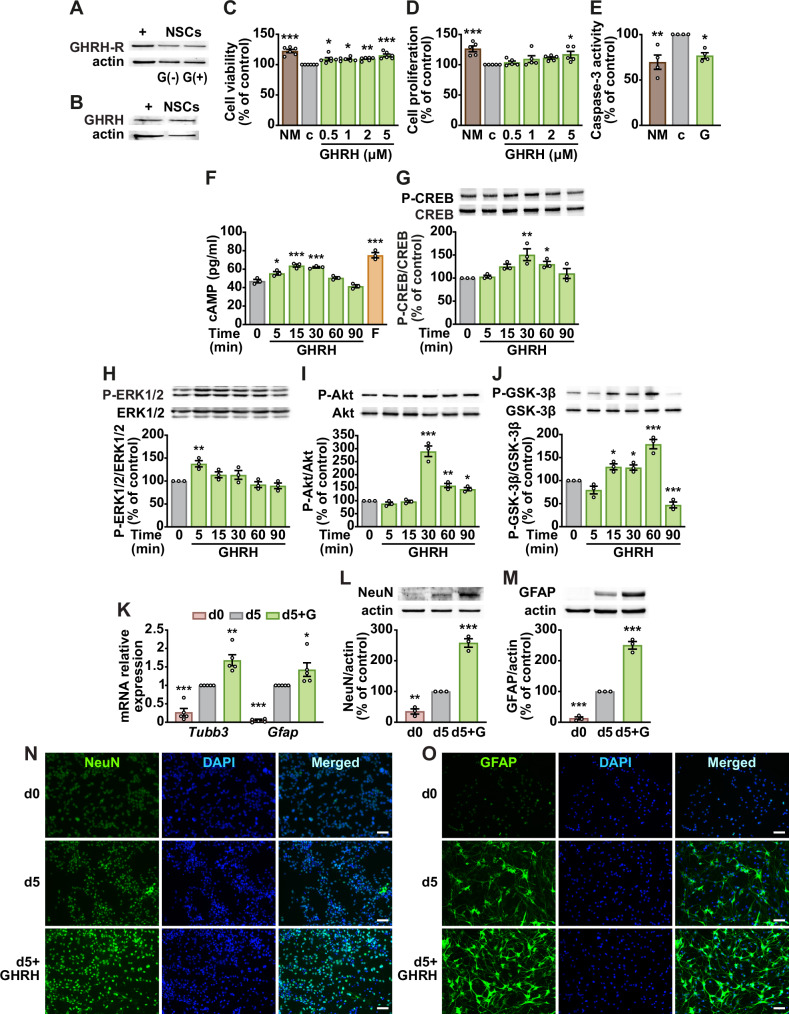
GHRH enhances neurogenesis and promotes pro-survival signaling in NSCs. Representative Western blot showing expression of GHRH-R (47 kDa) in NSCs cultured for 24 h without (–) or with (+) GHRH(1-44)NH_2_ (G, 5 μM) (**A**) and GHRH (12 kDa) (**B**). H9c2 cells were used as a positive control (+), and actin as loading control (*n* = 3). Cell viability (**C**) and proliferation (**D**) were assessed by MTT and BrdU assays, respectively, in NSCs cultured for 24 h in normal medium (NM) or growth factor-deprived medium (c, control), alone or with GHRH, at the indicated concentrations. Results, expressed as a percentage of control, are means ± SEM. *P < 0.05, **P < 0.01, ***P < 0.001 vs. c, one-way ANOVA with Dunnett’s post-hoc test (*n* = 6 for **C,** *n* = 5 for **D**). **E** Caspase-3 activity in NSCs cultured for 24 h in NM or in control medium (c), without or with GHRH (5 μM). Data, expressed as percentage of control, are means ± SEM. *P < 0.05, **P < 0.01 vs. c, one-way ANOVA with Tukey’s post-hoc test (*n* = 4). **F** Intracellular cAMP levels, measured by ELISA, in NSCs treated with GHRH (5 μM) for the indicated times. Forskolin (F, 50 μM for 2 min) served as positive control (*n* = 3). Representative Western blots of phosphorylated (P)-CREB (**G**), P-ERK1/2 (**H**), P-Akt (**I**), and P-GSK-3β (Ser9) (**J**) (top panels), in NSCs treated with GHRH (5 μM) for the indicated times. Results, normalized to respective nonphosphorylated proteins (bottom panels), are expressed as a percentage of control. Means ± SEM, *P < 0.05, **P < 0.01, ***P < 0.001 vs. time 0, one-way ANOVA with Dunnett’s post hoc test (*n* = 3). NSCs were cultured in differentiation medium for the indicated days (d), without or with GHRH (**G**) (5 μM). **K** Real-time PCR analysis for neuronal-specific β-tubulin isoform (*Tubb3*) and glial fibrillary acidic protein (*Gfap*). Results are normalized to *Rn18s* (*n* = 5). Representative Western blots for NeuN (**L**) and GFAP (**M**) (*top panels*). Results, normalized to actin (*bottom panels*), are expressed as a percentage of control (*n* = 3). Means ± SEM, *P < 0.05, **P < 0.01, ***P < 0.001 vs. day 5, one-way ANOVA with Tukey’s post-hoc test. Representative fluorescence microscopy images of NeuN (**N**) and GFAP (**O**) stained in green. Nuclei were counterstained with DAPI (blue). Scale bar: 50 μm.

Western blot analysis showed expression of GHRH-R and GHRH in NSCs, with no effect of GHRH stimulation on GHRH-R levels (Fig. 1A, B). Treatment with GHRH for 24 h in growth factor-deprived medium increased cell viability at all tested concentrations (0.5–5 μM), with the greatest effect at 5 μM (Fig. 1C). After 48 h, the GHRH-induced protection was slightly reduced and observed at 1–5 μM (Supplementary Fig. 1). Similarly, 5 μM GHRH promoted proliferation at 24 h, while lower doses showed a non-significant effect (Fig. 1D). GHRH-induced survival and proliferation were blocked by GHRH antagonist JV-1-36, suggesting receptor-dependent mechanisms (Supplementary Fig. 2A, B). Based on these results, 5 μM was chosen for subsequent experiments.

Growth factor deprivation increased caspase-3 activity, indicating elevated apoptosis, which was suppressed by GHRH exposure (Fig. 1E). GHRH also elevated intracellular cAMP levels at 5, 15, and 30 min, with a subsequent decline (Fig. 1F), and increased CREB phosphorylation at Ser133, which peaked at 30 and 60 min and decreased at 90 min (Fig. 1G). GHRH-induced increases in cell viability and proliferation were abolished by cAMP-pathway inhibitors, NF449 (Gαs antagonist) (Supplementary Fig. 2C, D), MDL-12330A (adenylyl cyclase inhibitor) (Supplementary Fig. 2E, F), and KT5720 (PKA inhibitor) (Supplementary Fig. 2G, H). GHRH also promoted phosphorylation of extracellular signal-regulated kinase (ERK)1/2, Akt, and GSK-3β, peaking at 5, 30, and 60 min (Fig. 1H–J), and ERK1/2 and PI3K/Akt inhibition with PD98059 and wortmannin, blocked its survival and proliferative effects (Supplementary Fig. 2I–L), whereas the inhibitors alone had no effect (Supplementary Fig. 2A–L). These findings collectively indicate that GHRH promotes survival and proliferation through cAMP/PKA/CREB as well as ERK1/2 and PI3K/Akt signaling pathways.

We next examined the effect of GHRH on NSC differentiation into neurons and astrocytes. The mRNA levels of the neuronal marker β-tubulin isoform (*Tubb3*) and the astrocyte marker glial fibrillary acidic protein (*Gfap*) increased by day 5 compared with day 0 (Fig. 1K) in differentiation medium, and this was accompanied by higher NeuN and GFAP protein expression (Fig. 1L, M). GHRH treatment further elevated both neuronal and astrocytic markers at the mRNA and protein levels (Fig. 1K–M), which was supported by stronger NeuN and GFAP immunofluorescence staining (Fig. 1N, O). Expression of the stem cell marker nestin declined during differentiation and was further reduced by GHRH (Supplementary Fig. 3). These findings suggest that GHRH enhances neurogenesis in vitro by promoting survival, proliferation, and lineage-specific differentiation.

### GHRH attenuates Aβ toxicity by modulating apoptotic, inflammatory, and neuroprotective pathways

**Fig. 2:**
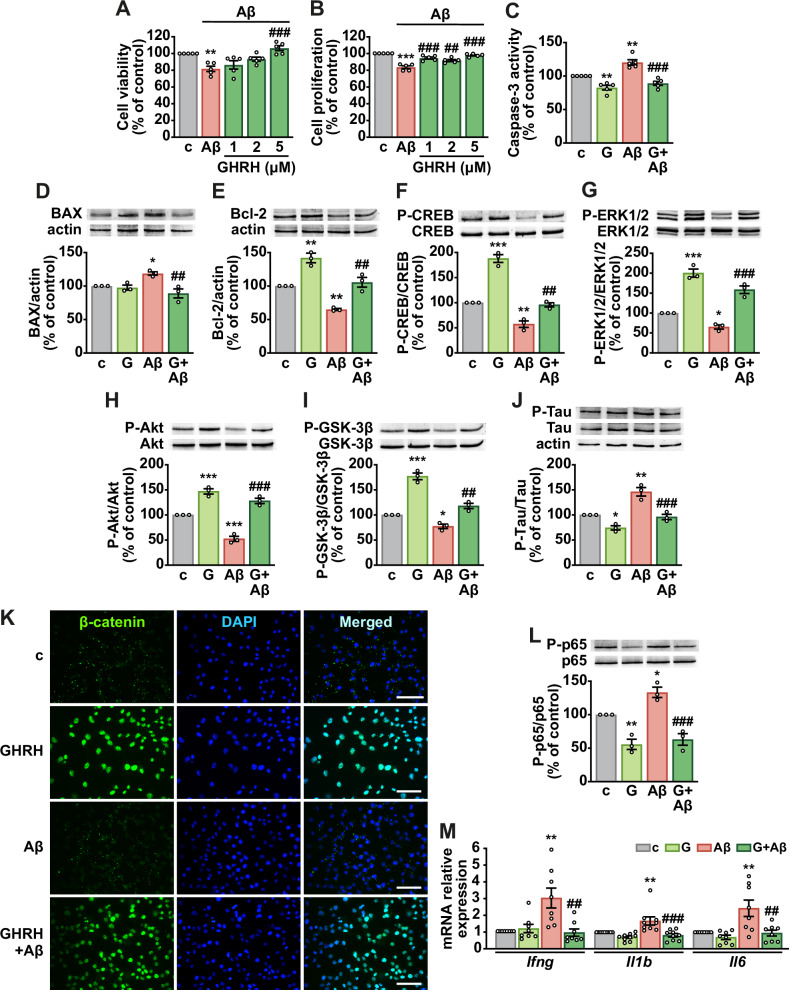
GHRH counteracts Aβ-induced toxicity in NSCs. Cell viability and proliferation assessed by MTT (**A**) and BrdU (**B**) assays in NSCs cultured for 24 h in control medium (c) with or without Aβ₁₋₄₂ (200 nM), alone or in combination with GHRH at the indicated concentrations. **C** Apoptosis evaluated by caspase-3 activity GHRH was used at 5 μM. Results for (**A**–**C**) are expressed as percentage of control (*n* = 5). Representative Western blots for BAX (**D**), Bcl-2 (**E**), phosphorylated (P)-CREB (**F**), P-ERK1/2 (**G**), P-Akt (**H**), P-GSK-3β (Ser9) (**I**), and P-Tau (Ser396) (**J**) (top panels). Actin (**D, E**) or nonphosphorylated proteins (**F**–**J**) served as loading control; actin served as internal control in **J** (bottom panels) (*n* = 3). **K** Representative fluorescence micrographs of β-catenin staining (green). Nuclei were counterstained with DAPI (blue). Scale bar: 50 μm. **L** Representative Western blot for phosphorylated (P)-p65 (*top*). Nonphosphorylated protein served as loading control (*bottom*) (*n* = 3). **M** mRNA expression of interferon-gamma (*Ifng)*, interleukin-1 beta (*Il1b*) and interleukin-6 (*Il6*) assessed by real-time PCR. Results, normalized to *Rn18s*, are expressed as fold-change relative to control (c) (*n* = 9). Data are means ± SEM. *P < 0.05, **P < 0.01, ***P < 0.01 vs. c; ^##^*P* < 0.01, ^###^*P* < 0.001 vs. Aβ, one-way ANOVA with Tukey’s post hoc test.

The protective effects of GHRH were assessed in NSCs exposed to Aβ_1-42_. Aβ (50-2000 nM) reduced cell viability in a dose-dependent manner over 24 h, with 200 nM chosen for subsequent experiments (Supplementary Fig. 4). GHRH at 5 μM reversed Aβ-induced decrease of cell viability and restored proliferation at all tested concentrations (1-5 μM) (Fig. 2A, B), effects that were abolished by JV-1-36 (Supplementary Fig. 5A, B). GHRH also reduced apoptosis by lowering caspase-3 activity (Fig. 2C), decreasing proapoptotic BAX (Fig. 2D), and increasing the antiapoptotic protein Bcl-2 (Fig. 2E). Mechanistically, GHRH counteracted Aβ-induced reductions in CREB, ERK1/2, and Akt phosphorylation and exerted similar effects even without Aβ exposure (Fig. 2F–H). The involvement of these pathways was confirmed by the ability of NF449, MDL-12330A, KT5720 (Supplementary Fig. 5C-H), and PD98059 or wortmannin (Supplementary Fig. 5I-L) to block GHRH-induced survival and proliferation. GHRH also reversed Aβ-mediated phosphorylation of GSK-3β (Fig. 2I) and tau (Fig. 2J). Furthermore, it enhanced nuclear β-catenin staining (Fig. 2K), reduced p65 NF-κB phosphorylation (Fig. 2L), and downregulated the proinflammatory cytokines interferon-gamma *(Ifng*), interleukin-1 beta (*Il1b*) and interleukin-6 (*Il6*) (Fig. 2M). Together, these findings indicate that GHRH protects NSCs from Aβ-induced cell death and apoptosis by attenuating tau phosphorylation and suppressing key AD-related pathogenic pathways.

### GHRH enhances neurogenesis and reduces Aβ toxicity in human neuroblastoma and neuron-like cells

**Fig. 3:**
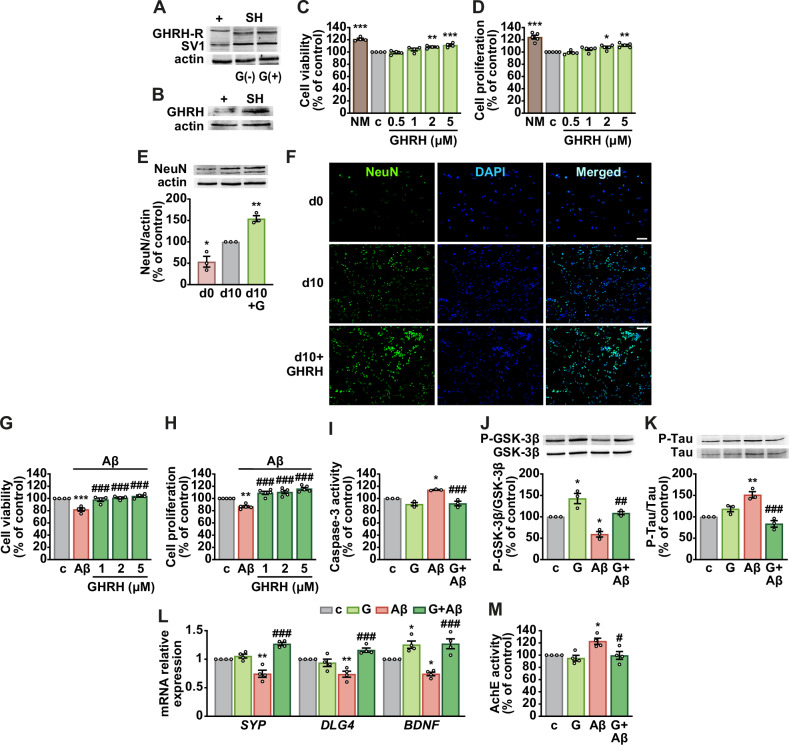
Neurogenic and neuroprotective effects of GHRH in human SH-SY5Y cells. Representative Western blot showing expression of GHRH-R (47 kDa) and SV1 (39.5 kDa) (*top*) in cells cultured for 24 h without (-) or with (+) GHRH (G, 5 μM) (**A**) and GHRH (**B**). PC-3 human prostate cancer cells served as positive control (+) and actin as loading control (*n* = 3). Cell viability (**C**) and proliferation (**D**) by MTT and BrdU assays, respectively, in cells cultured for 24 h in normal medium (NM) or growth factor-deprived medium (c), without or with GHRH at the indicated concentrations. Results, expressed as a percentage of control, are means ± SEM. *P < 0.05, **P < 0.01, ***P < 0.001 vs. c, one-way ANOVA with Dunnett’s post-hoc test (*n* = 4 for **C,** *n* = 5 for **D**). **E** Representative Western blot for NeuN (*top*) in cells cultured in differentiation medium for the indicated days (d), with or without GHRH (5 μM). Results, normalized to actin, are expressed as a percentage of day 10. Means ± SEM. *P < 0.05, **P < 0.01 vs. d10, one-way ANOVA with Tukey’s post-hoc test (*n* = 3). **F** Representative fluorescence micrographs of NeuN (green). Nuclei were counterstained with DAPI (blue) (scale bar: 50 μm**)**. Cell viability (**G**) and proliferation (**H**) by MTT and BrdU, respectively, in SH-SY5Y cells cultured in control medium (c) for 24 h with or without Aβ_1-42_ (10 μM), alone or with GHRH at the indicated concentrations or 5 μM (*n* = 4 for **G,** *n* = 5 for **H**). **I** Apoptosis assessed by caspase-3 activity (*n* = 3). Representative Western blots for P-GSK-3β (Ser9) (**J**) and P-Tau (**K**) (top panels). Nonphosphorylated proteins served as loading control (bottom panels) (*n* = 3). **L** Real-time PCR analysis for synaptophysin (*SYP*), PSD95 (*DLG4*) and BDNF, normalized to Rn18s in differentiated SH-SY5Y cultured in control medium (c) for 24 h, with or without Aβ_1-42_ (10 μM), alone or with GHRH (5 μM) (*n* = 4). **M** Acetylcholinesterase (AChE) activity in differentiated SH-SY5Y (*n* = 4). Data are means ± SEM. *P < 0.05, **P < 0.01, ***P < 0.001 vs. c; ^*#*^*P* < 0.05, ^*##*^*P* < 0.01, ^*###*^*P* < 0.001 vs. Aβ, one-way ANOVA with Tukey’s post hoc test.

The effects of GHRH were further investigated in SH-SY5Y cells, a widely used in vitro neuronal model capable of differentiating into mature neuron-like cells [29]. Western blotting confirmed the expression of GHRH-R, its splice variant SV1 (Fig. 3A), and GHRH itself (Fig. 3B). In undifferentiated cells, 24 h GHRH treatment increased viability and proliferation in a dose-dependent manner and counteracted growth factor deprivation, particularly at 2 and 5 μM (Fig. 3C, D). During differentiation induced by all-trans retinoic acid (RA) and brain-derived neurotrophic factor (BDNF), GHRH elevated NeuN expression (Fig. 3E, F) and reduced nestin levels (Supplementary Fig. 6). Dose-response analysis (Supplementary Fig. 7) showed that Aβ_1-42_ (10 μM) decreased cell viability and proliferation, whereas GHRH (1-5 μM) reversed these effects (Fig. 3G, H). GHRH also suppressed Aβ-induced caspase-3 activation (Fig. 3I), restored GSK-3β phosphorylation (Fig. 3J), and lowered tau phosphorylation (Fig. 3K). In SH-SY5Y-derived neuron-like cells, GHRH counteracted Aβ-induced reductions in synaptophysin, PSD95 (DLG4), and BDNF mRNA levels, while also upregulating *BDNF* on its own (Fig. 3L). GHRH further prevented the Aβ-induced increase in acetylcholinesterase (AChE) activity (Fig. 3M). Together, these findings indicate that GHRH promotes neurogenesis and mitigates Aβ toxicity in undifferentiated cells, while supporting neuronal and synaptic function in neuron-like cells.

### MR-409 reduces Aβ plaque load and tau phosphorylation in 5xFAD mice

**Fig. 4:**
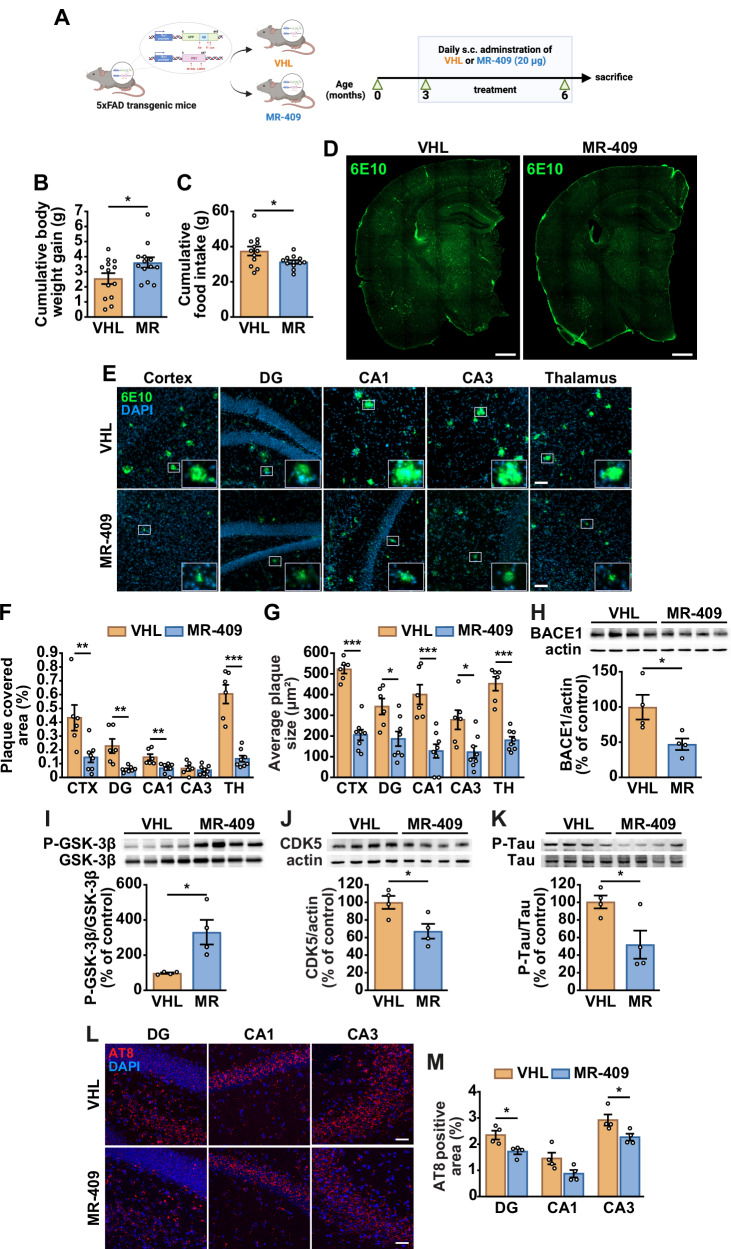
Effect of MR-409 on body weight, food intake, Aβ deposition and Tau phosphorylation in 5xFAD mice. **A** Schematic representation of the treatment protocol (created with Biorender.com). Body weight gain (**B**) and food intake (**C**) (*n* = 13 for **B**, and 12 for **C**). **D** Representative Aβ immunostaining with 6E10 antibody (green) in brain sections. Scale bar: 1000 μm. **E** Representative 6E10 immunostaining (green) in the cortex, hippocampus [dentate gyrus (DG), CA1, CA3], and thalamus. Nuclei were counterstained with DAPI (blue). Insets show magnified plaques. Scale bar: 50 μm. Quantification of plaque covered area (**F**) and average plaque size (**G**) (VHL, *n* = 6; MR-409, *n* = 8). Representative Western blots for BACE1 (**H**), P-GSK-3β (**I**), CDK5 (**J**), and P-Tau (**K**) (*top panels*) in brain lysates. Actin (**H, J**) or nonphosphorylated proteins (**I, K**) served as loading controls (*bottom panels*). Results are expressed as percentage of control (VHL) (*n* = 4 per group). **L** Representative AT8 immunostaining for p-tau in the indicated brain regions. Nuclei were counterstained with DAPI (blue). Scale bar: 100 μm. **M** Quantification of AT8-positive area (*n* = 4 per group). Data are means ± SEM. *P < 0.05, **P < 0.01, ***P < 0.001 vs. VHL, unpaired Student’s t-test.

To investigate the protective effects of GHRH in vivo, 3-month-old male 5xFAD mice were treated subcutaneously with GHRH agonist MR-409 (20 μg) or vehicle (Fig. 4A). These mice exhibit early amyloid accumulation in the brain, microglial activation and neuroinflammation [30, 31]. MR-409-treated mice showed increased body weight (Fig. 4B) and decreased food intake compared with vehicle controls (Fig. 4C). MR-409 decreased Aβ plaque deposition, as shown by 6E10 immunofluorescence (Fig. 4D), particularly in the cortex, hippocampal dentate gyrus (DG) and CA1, and thalamus (Fig. 4E, F). Correspondingly, the total plaque area was reduced in all regions analysed (Fig. 4G). These effects extended to the anterior cingulate cortex (ACC), retrosplenial cortex (RSP), motor cortex and somatosensory cortex, but were absent in the striatum (Supplementary Fig. 8). The β-secretase BACE1, which drives Aβ production by cleaving APP, is a central contributor to AD pathology [7, 32]. Western blot analysis showed that MR-409 reduced BACE1 levels relative to vehicle-treated mice (Fig. 4H), implying decreased APP cleavage and Aβ formation.

Kinases such as GSK-3β and cyclin-dependent kinase 5 (CDK5) drive tau hyperphosphorylation [2, 7]. MR-409 increased inhibitory GSK-3β phosphorylation at Ser9 (Fig. 4I) and reduced CDK5 expression (Fig. 4J), thereby lowering tau phosphorylation (Fig. 4K). Consistently, AT8 immunostaining showed decreased tau phosphorylation at Ser202/Thr205 in the DG and CA3 regions, with a similar trend in CA1 (Fig. 4L, M). These results indicate that MR-409 mitigates Aβ accumulation and tau phosphorylation in 5xFAD mice.

### MR-409 attenuates microglial and astrocyte activation in 5xFAD mice

**Fig. 5:**
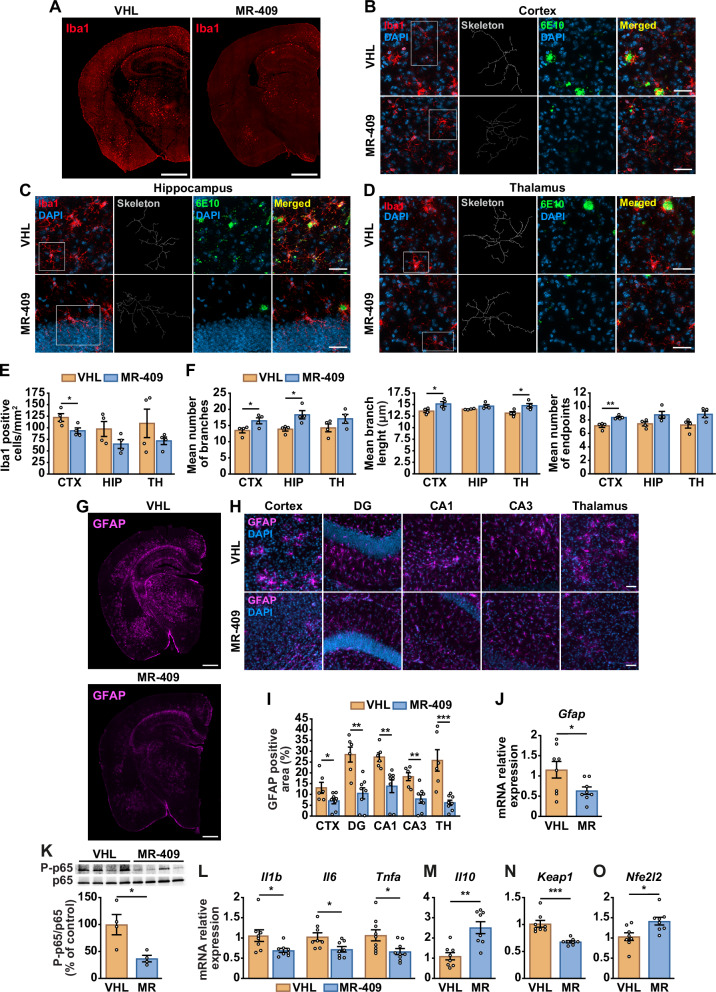
Attenuation of microgliosis and astrogliosis by MR-409 in 5xFAD mice. Mice were treated with vehicle (VHL) or MR-409 (20 μg). **A** Representative immunostaining for microglia with Iba1 antibody (red) in brain sections of mice treated with VHL or MR-409. Scale bar: 1000 μm. Representative images of Iba1-labeled microglia (red) and 6E10-positive Aβ plaques (green) in the cortex (**B**) hippocampus (**C**) and thalamus (**D**). Nuclei were counterstained with DAPI (blue). Skeletonized cells are shown for morphological analysis. Scale bar: 50 μm. **E** Quantification of Iba1-positive cells in the cortex (CTX), hippocampus (HIP), and thalamus (TH) (*n* = 4 per group). **F** Morphological analysis of individual microglial cells, including mean number of branches, mean branch length and mean number of endpoints (*n* = 4 per group). **G** Representative GFAP immunostaining for astrocytes (magenta) in brain sections of mice treated with VHL (*top*) or MR-409 (*bottom*). Scale bar: 1000 μm. **H** Representative GFAP immunostaining (magenta) in the cortex, hippocampus [dentate gyrus (DG), CA1, CA3], and thalamus. Nuclei were counterstained with DAPI (blue). **I** Quantification of GFAP-positive area in the indicated brain regions (VHL, *n* = 6; MR-409, *n* = 8). **J** Real-time PCR analysis for *Gfap* mRNA normalized to *Rn18s* in brain lysates (*n* = 8 per group). **K** Representative Western blot for phosphorylated p65 (P-p65) in brain lysates. Results, normalized to nonphosphorylated protein are expressed as percentage of control (VHL) (*n* = 4 per group**)**. **L-O** Real-time PCR analysis for the following genes: interleukin-1 beta (*Il1b*), interleukin-6 (*Il6*) and tumor necrosis factor-alpha (*Tnfa*) (**L**); interleukin-10 (*Il10*) (**M**), *Keap1* (**N**), and *Nfe2l2* (NRF2) (**O**). Results, normalized to *Rn18s*, are expressed as fold-change relative to control (*n* = 8 per group). Data are means ± SEM. *P < 0.05, **P < 0.01, ***P < 0.001 vs. VHL, unpaired Student’s t-test.

Neuroinflammation, largely driven by microglia and astrocytes, is a major contributor to AD pathogenesis [2, 3]. To evaluate the anti-inflammatory effects of MR-409 in 5xFAD mice, we assessed microglial activation by immunostaining for ionizing calcium-binding adaptor molecule 1 (Iba1). MR-409-trated mice showed reduced brain Iba1 immunoreactivity compared with vehicle controls (Fig. 5A). Specifically, MR-409 decreased both Iba1 staining and Aβ plaque load, detected by 6E10, in the cortex, hippocampus, and thalamus (Fig. 5B-D). A significant reduction in Iba1-positive cells was observed in the cortex, with a similar downward trend in the hippocampus and thalamus (Fig. 5E). Morphological analysis revealed features consistent with reduced microglial activation, including increased ramification in the cortex and hippocampus, greater branch length in the cortex and thalamus, and a higher number of endpoints in the cortex, with comparable trends in the hippocampus and thalamus (Fig. 5F). Astrogliosis was likewise attenuated in MR-409-treated brains, as shown by reduced GFAP immunoreactivity relative to vehicle controls (Fig. 5G). Significant decreases were detected in the cortex, DG, CA1, CA3, and thalamus (Fig. 5H, I), accompanied by overall downregulation of *Gfap* expression (Fig. 5J). GFAP levels were also reduced in the striatum and retrosplenial cortex (RSP), but not in the ACC, motor cortex, or somatosensory cortex (Supplementary Fig. 9). Consistent with diminished glial activation, MR-409 decreased NF-κB signaling, evidenced by reduced p65 phosphorylation (Fig. 5K), downregulated the proinflammatory cytokines *Il1b, Il6*, and *Tnfa*, while increasing the anti-inflammatory cytokine *Il10* (Fig. 5L, M). Because NRF2 protects against oxidative stress and Aβ pathology in AD and is negatively regulated by KEAP1 [33], we next examined NRF2 signaling. MR-409 treatment decreased *Keap1* expression (Fig. 5N) and upregulated *Nfe2l2* (NRF2) (Fig. 5O) in 5xFAD mice, suggesting activation of the NRF2 pathway. Together, these findings demonstrate that MR-409 mitigates neuroinflammation in 5xFAD mice by limiting gliosis, suppressing proinflammatory mediators, and enhancing oxidative stress defences.

### MR-409 mitigates neuronal loss and improves cognitive functions in 5xFAD mice

**Fig. 6:**
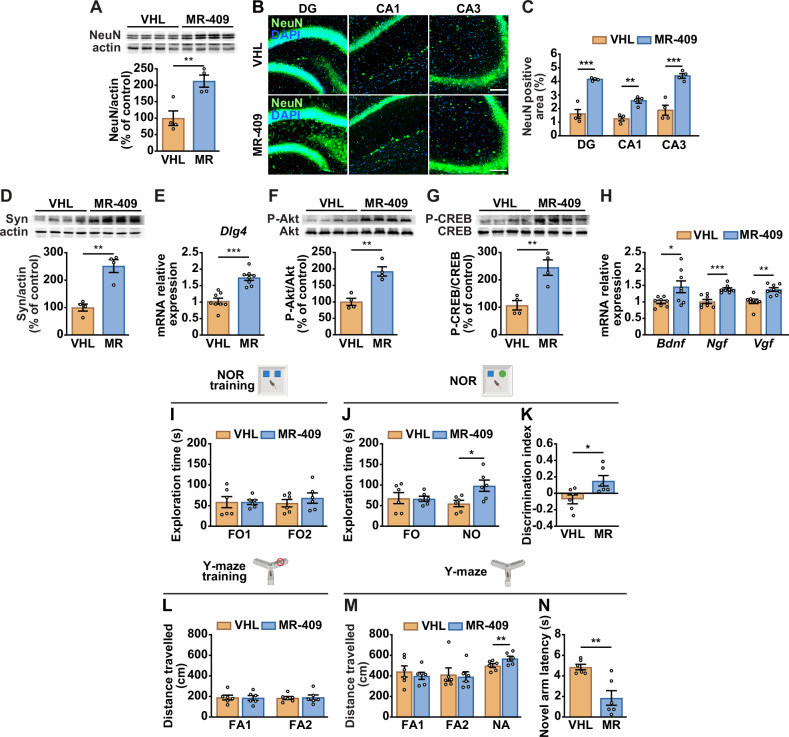
Effect of MR-409 on neuronal loss and cognitive impairment. 5xFAD mice were treated with vehicle (VHL) or MR-409 (20 μg). **A** Representative Western blot for NeuN (*top panel*) in brain lysates. Results, normalized to actin (*bottom panel*), are expressed as percentage of control (vehicle, VHL) (*n* = 4 per group). **B** Representative NeuN immunostaining in the hippocampus (DG, CA1 and CA3 regions). Nuclei were counterstained with DAPI (blue). Scale bar: 100 μm. **C** Quantification of NeuN-positive area (*n* = 4 per group). **D** Representative Western blot for synaptophysin (Syn) in brain lysates. Results are expressed as percentage of control (VHL) (*n* = 4 per group). **E** Real-time PCR analysis for *Dlg4*, normalized to *Rn18s* (*n* = 8 per group). Representative Western blot for phosphorylated (P)-Akt (**F**) and P-CREB (**G**) (*top panels*) in brain lysates. Results, normalized to nonphosphorylated proteins (*bottom panels*), are expressed as a percentage of control (VHL) (*n* = 4 per group). **H** Real-time PCR analysis for *Bdnf*, *Ngf*, and *Vgf*, normalized to *Rn18s* (*n* = 8 per group). Novel object recognition (NOR) test. Exploration time, expressed in seconds (s), of the two identical familiar objects (FO1 and FO2) during the training phase (**I**) and of the familiar (FO) and novel (NO) objects during the test phase (**J**). **K** Discrimination index. Y-maze test. Distance traveled, expressed in centimeters (cm), into the two familiar (FA1 and FA2) arms during the training phase (**L**) and into the two familiar (FA1 and FA2) arms and the novel (NA) arm during the test phase (**M**). **N** Latency to enter the novel arm, expressed in seconds (s). Data are means ± SEM. *P < 0.05, **P < 0.01, ***P < 0.001 vs. VHL, unpaired Student’s t-test or one-way repeated-measures ANOVA with Tukey’s post hoc test (for **M**).

Neuronal loss, together with Aβ accumulation and tau hyperphosphorylation, is a hallmark of AD and is also evident in 5xFAD mice [30, 34]. MR-409 treatment increased NeuN protein levels in 5xFAD brains compared with vehicle-treated controls (Fig. 6A), and immunostaining revealed stronger NeuN signal in the DG, CA1, and CA3 regions (Fig. 6B, C), indicating improved neuronal survival. MR-409 also preserved synaptic integrity, evidenced by elevated synaptophysin (Fig. 6D) and *Dlg4* (Fig. 6E). In addition, MR-409 enhanced Akt and CREB phosphorylation (Fig. 6F, G), and upregulated *Bdnf*, nerve growth factor (*Ngf*), and *Vgf* expression (Fig. 6H). Notably, MR-409 did not alter plasma GH or IGF1 levels (Supplementary Fig. 10).

Memory deficits in 5xFAD mice generally appear by 4-5 months of age [30]. To assess whether MR-409 affects cognitive performance, we conducted novel object recognition (NOR) and Y-maze tests. No significant differences in basal exploratory activity during the training sessions of both tests were observed between vehicle- and MR-409-treated 5xFAD mice (Fig. 6I, L). However, while exploration of the familiar object was comparable between groups, MR-409-treated mice spent more time exploring the novel object and exhibited a higher discrimination index, reflecting improved recognition memory (Fig. 6J, K). In the Y-maze, total distance traveled in the familiar arms did not differ; nevertheless, MR-409-treated mice traveled farther in the novel arm (Fig. 6M) and entered it more rapidly (Fig. 6N), indicating enhanced spatial memory.

Collectively, these findings indicate that MR-409 promotes neuroprotection by activating neuroprotective and neurotrophic pathways, thereby improving memory deficits in 5xFAD mice.

## Discussion

This study shows that GHRH and its agonist MR-409 exert both neurogenic and neuroprotective effects in AD models. GHRH(1-44)NH₂ promoted survival, proliferation and differentiation of rat NSCs and human neuroblastoma cells, while preserving synaptic integrity and cholinergic function in human neuron-like cells exposed to Aβ. In 5xFAD mice, MR-409 reduced Aβ accumulation and tau phosphorylation, attenuated neuroinflammation and oxidative stress, protected against neuronal and synaptic loss, and improved memory performance.

Adult hippocampal neurogenesis (AHN), which occurs primarily in the dentate gyrus, involves the proliferation, differentiation, and integration of NSCs into hippocampal circuits essential for learning and memory. Although the extent of AHN in humans remains debated, it is reduced in AD, underscoring its potential as a therapeutic target. Notably, NSCs persist throughout aging and diminished AHN has been associated with cognitive decline, potentially representing an early hallmark of AD progression [35]. Here, we show that NSCs express both GHRH and its receptor, indicating potential autocrine or paracrine activity; however, the lack of response to the JV-1-36 antagonist suggests that endogenous signaling is limited. In contrast, exogenous GHRH enhanced NSC viability, proliferation, and resistance to apoptosis under growth factor deprivation and Aβ exposure, consistent with prior studies [36, 37]. Mechanistically, GHRH activated the cAMP/PKA/CREB pathway even in the presence of Aβ, in line with canonical GHRH-R signaling [38]. Since CREB, an essential regulator of neurogenesis and synaptic plasticity through the Ras/MAPK and PI3K/Akt pathways, is reduced in AD [39], its restoration is likely beneficial. Accordingly, GHRH activated PI3K/Akt and ERK1/2 signaling, both known to counteract Aβ toxicity [40] and inhibition of these pathways diminished its survival and proliferative effects of GHRH, confirming their central contribution. Although ERK can be aberrantly activated in AD [41], our data suggest that its phosphorylation supports GHRH-induced mitogenesis, consistent with previous reports [38]. Because these pathways also underlie neurotrophin-driven neurogenesis [41, 42], the ability of GHRH to promote NSC differentiation into neurons and astrocytes support its neurogenic potential.

Aβ oligomers impair NSC function by reducing proliferation and neurogenesis while promoting gliogenesis and inflammation, through activation of GSK-3β and suppression of β-catenin signaling. GSK-3β further drives tau hyperphosphorylation and Aβ accumulation, whereas its inhibition enhances NSC survival [43]. Conversely, the Wnt/β-catenin pathway promotes neurogenesis and protection against Aβ toxicity [8]. In our study, GHRH attenuated Aβ-induced NSC dysfunction by decreasing tau phosphorylation, elevating nuclear β-catenin, and reducing proinflammatory cytokine expression. Likewise, in SH-SY5Y-derived neuron-like cells, GHRH prevented Aβ-induced decreases in synaptic markers and BDNF, and attenuated the Aβ-driven increase in AChE activity, indicating preserved synaptic integrity and cholinergic function [7].

Consistent with our findings, MR-409 has been shown to promote neurogenesis in cerebral ischemia models and in NSCs by activating Akt, ERK, CREB, and BDNF/TrkB signaling [16]. Interestingly, these pathways mediate the proliferative effects of GH secretagogues, including hexarelin and ghrelin, on NSCs [44, 45]. In addition, we previously demonstrated that GHRH counteracts TNF-α-induced atrophy in C2C12 myotubes by activating PI3K/Akt and GSK-3β while suppressing NF-κB signaling [46]. NSC transplantation ameliorates cognitive impairments and pathological features in AD models, and its combination with pharmacological agents can produce synergistic benefits. benefits [47]. Given the neuroprotective properties of GHRH and MR-409 [11, 15, 16], additional research is needed to determine their potential to enhance NSC-based therapies for AD.

To assess its neuroprotective effects in vivo, GHRH was tested in 5xFAD transgenic mice, which recapitulate key features of AD [30, 31]. Male 5xFAD mice received subcutaneous administration of GHRH agonist MR-409 at 20 µg/day. Previous studies have shown efficacy of 5–15 µg/mouse/day subcutaneously in adult models without elevating serum GH [16, 48], while neonatal protocols have used much higher doses of 1–2 mg/kg/day [15]. The 20 µg/day dose used here (≈0.67–1.0 mg/kg/day) is above the typical adult range but far below neonatal levels, providing adequate tissue penetration without activating the GH axis. Accordingly, MR-409 did not change circulating GH or IGF1 levels in 5xFAD mice, consistent with previous findings in other models [36, 49, 50]. Although MR-409 may promote a transient GH rise, sustained levels remain unchanged [25]. In cancer models, MR-409 even lowers IGF1 [50], indicating tissue-specific actions rather than broad GH/IGF1 axis activation. This could be beneficial, since systemic IGF1 is associated with oncogenic risk, whereas a brain-targeted mechanism could offer a safer alternative.

Weight loss is frequently observed in both AD patients and 5xFAD mice, primarily attributed to medications, hypercortisolism and muscle wasting in humans and to hypermetabolism in mice [51, 52]. Here, MR-409-treated mice gained more weight than controls despite reduced food intake, consistent with previous findings in SMA mice and diabetic retinopathy models [15, 28]. Further research is needed to elucidate the role of GHRH agonists in weight regulation.

Aβ accumulation drives AD by disrupting the balance between its production and clearance, subsequently triggering tau tangles, inflammation, oxidative stress, and mitochondrial dysfunction, culminating in synaptic and neuronal loss [5, 53]. Memory-related regions like the hippocampus, cortex and thalamus are particularly vulnerable [54]. In 5xFAD mice, plaques appear in the hippocampus and cortex by 2 months of age, extending to the thalamus by 6 months [30, 31, 54]. MR-409 treatment significantly reduced Aβ burden and plaque size in these regions, with a decrease in BACE1 expression, highlighting the peptide’s potential to counteract AD-related neurodegeneration.

GSK-3β and CDK5 are major drivers of AD pathology, promoting Aβ accumulation, tau hyperphosphorylation, and neuroinflammation [7, 55]. In 5xFAD mice, MR-409 lowered GSK-3β activity and CDK5 expression, reducing hippocampal tau phosphorylation and suggesting a direct impact on tau pathology. Given that hyperphosphorylated tau disrupts microtubule stability and amplifies inflammation and oxidative stress [2, 3, 33], the ability of MR-409 to attenuate microgliosis, decrease GFAP across multiple regions, and suppress NF-κB-mediated cytokine production, suggest broad anti-inflammatory effect. These results align with previous observations in SMA mice [15] and ischemic stroke models [16]. MR-409 also enhanced antioxidant defenses by upregulating Nrf2 and decreasing Keap1, similar to responses observed in diabetic retinopathy [28] and with the GHRH agonist JI-34 in hypoxic conditions [26], supporting a role for GHRH analogs in mitigating oxidative stress-driven neurodegeneration.

Neuronal and synaptic loss, largely induced by Aβ pathology, is well documented in 5xFAD mice by 6 months of age [31, 34, 56]. In our study, MR-409 improved neuronal integrity, as indicated by increased hippocampal NeuN staining, a standard marker for neuronal survival [57], and supported synaptic preservation, evidenced by elevated synaptophysin and PSD95 mRNA levels. MR-409 also enhanced Akt and CREB phosphorylation and upregulated neurotrophins such as BDNF, NGF, and VGF, which are typically reduced in AD [42, 58]. These molecular and histological changes were accompanied by improved recognition and spatial memory, suggesting that the cellular benefits may translate into functional outcomes. Together with similar findings in stroke models [16], these results suggest that MR-409 mitigates multiple aspects of AD pathology and supports neuronal and synaptic integrity through neurotrophic and pro-survival pathways.

GHRH analogs, as well as GHRH and sermorelin, the parent peptide of MR-409, have been shown to cross the blood-brain barrier (BBB) [59–61], although direct pharmacokinetic evidence for MR-409 is still lacking. Notably, most neuroprotective actions of MR-409 have been observed in models with BBB disruption, where CNS entry is more feasible [15, 16, 28, 62]. Given that BBB breakdown is a recognized feature of AD, including in 5xFAD mice [63, 64], direct central activity of MR-409 remains plausible. However, MR-409 also exerts systemic metabolic, anti-inflammatory, and vascular effects, suggesting that its benefits in 5xFAD mice may reflect combined central and peripheral mechanisms. The absence of in vivo receptor-occupancy data makes it difficult to determine how much MR-409 engages brain GHRH receptors. As a result, its activity is inferred indirectly from upstream signaling changes (cAMP/PKA-CREB, AKT/CREB, BDNF/TrkB) and from downstream effects such as increased neurogenesis, vascular and renal protection, and β-cell preservation [25].

In contrast with our findings, the GHRH antagonist MIA-690 has previously shown protective effects in 5xFAD mice [65]. This discrepancy likely reflects differences in dosing and the context-dependent nature of GHRH signaling rather than contradictory pharmacology. MIA-690 was administered at 2-10 µg/day from 3-9 months of age, whereas MR-409 was administered at 20 µg/day from 3-6 months. Because agonists and antagonists act through distinct mechanisms, their effective doses are not directly comparable: MIA-690 primarily reduces IGF1, while MR-409 activates survival pathways, decreases GSK-3β-dependent tau phosphorylation, and suppresses inflammation and oxidative stress without altering systemic GH/IGF1. Despite opposite receptor actions, both engage GHRH-R/SV1 and may converge on protective signaling pathways, highlighting the need to explore potential biased or tissue-specific effects. Notably, MR-409, but not the antagonist MIA-602, protects against diabetic retinopathy [28], underscoring functional differences among GHRH analogs. GHRH peptides may also share features with the GH-secretagogue ghrelin, which activates hippocampal neurons and enhances spine formation and spatial memory [66, 67].

MR-409 exhibits broad CNS benefits in aging, including neuroprotection, enhanced plasticity, and reduced neuroinflammation. Accordingly, subcutaneous administration of the long-acting GHRH analog tesamorelin has improved executive function in adults with mild cognitive impairment and in healthy older adults [21]. In various in vivo models, MR-409 is well tolerated at therapeutic doses, demonstrating cardioprotective [68] and antidiabetic effects [24], without consistent organ-specific toxicity. However, these data remain preclinical, and human pharmacokinetics and dose-limiting toxicities are still unknown. Although GHRH analogs are more stable than native GHRH [23], they undergo rapid gastrointestinal degradation, requiring daily intravenous or subcutaneous administration. New formulations, such as BSA/heparin nanoparticles for sustained release, may improve stability but require further development.

In summary, this study shows that GHRH exerts protective and neurogenic actions in vitro. In 5xFAD mice, MR-409 reduces amyloid-β accumulation, tau phosphorylation and neuroinflammation, while also mitigating neuronal and synaptic loss and improving cognitive functions. Although this study has several limitations, they do not reduce the overall significance of our findings. First, the use of 5xFAD mice, which primarily reflects amyloid-driven pathology, limiting direct extrapolation to sporadic AD or tauopathies, although they remain a robust model of early amyloid-related neurodegeneration [30, 31]. Second, the exclusive use of male mice may have masked potential sex-specific differences in disease progression or treatment responses; inclusion of both sexes will be important in future studies.

In conclusion, our findings identify GHRH agonists such as MR-409 as multifunctional modulators of AD-related pathology and support their further translational development as therapeutic candidates for neurodegenerative diseases.

## Materials and methods

### Cell culture and treatments

H9c2 rat cardiac cells (#CRL-1446, RRID:CVCL_0286) and PC3 human prostate cancer cells (#CRL-1435, RRID:CVCL_0035) (ATCC, Manassas, VA) were cultured as described previously [36]. Adul rat hippocampal NSCs (Sigma-Aldrich, #SCR022) were maintained in polyornithine (Sigma-Aldrich, #P4957) coated flasks or wells using Dulbecco′s Modified Eagle′s Medium (DMEM)/F12 supplemented with 2% B27 (ThermoFisher, #17504044), 20 ng/ml human basic fibroblast growth factor (b-FGF) (ThermoFisher, #PHG0021), 2 mM L-glutamine, 100 U/ml penicillin, 100 μg/ml streptomycin and 250 ng/mL amphotericin B (normal medium, NM) at 37 °C with 5% CO_2_. Cells were switched to DMEM/F12 with 0.1% bovine serum albumin (BSA), (Sigma-Aldrich, #A2153) without growth factors and b-FGF for 2 h (control medium, c), then exposed to the different stimuli. For differentiation, NSCs were cultured in medium containing 0.8% N2 (ThermoFisher Scientific, #17502048), 1% fetal bovine serum (FBS) and 1 μM retinoic acid (RA) (MedChemExpress, #HY-14649) for 5 days, with or without rat GHRH(1-44)NH_2_ (5 μM) (MyBioSource, #MBS826486) and medium was changed every 2 days. SH-SY5Y human neuroblastoma cells (IBVR, Italy, #BSTCL 232, RRID:CVCL_0019) were cultured in DMEM/F12 with 10% FBS and standard supplements. Cells were preconditioned in DMEM/F12 with 1% FBS before treatments. At confluence, differentiation was conducted for 10 days with or without human GHRH (5 μM) (MyBioSource, #MBS826402), 10 μM RA for 3 days, followed by RA and 50 ng/ml brain-derived neurotrophic factor (BDNF) (MedChemExpress, #HY-P7116A) on days 4 and 7 [29]. Cells were routinely tested for mycoplasma contamination using a PCR-based assay, and only mycoplasma-free cultures were used for experiments.

### Cell viability and proliferation

NSCs and SH-SY5Y cells were seeded in 96-well plates (10 × 10^3^ cells/well) in NM. Cell viability and proliferation were assessed by 2,5-diphenyl tetrazolium bromide (MTT) assay (Sigma-Aldrich, #M5655) and 5-bromo-2-deoxyuridine (BrdU) incorporation ELISA kit (Roche Diagnostic, #11647229001), respectively, as described [36, 46, 69].

### Caspase-3 activity

NSCs and SH-SY5Y cells were seeded in 6-well plates in NM (5 × 10⁵ cells per well). Caspase-3 activity in cell lysates was assessed by Caspase-3 Colorimetric Assay Kit (Abcam, #ab39401) following the manufacturer’s protocol. Briefly, cells were resuspended in Cell Lysis Buffer, incubated at 4 °C for 10 min, and centrifuged to collect cytosolic extracts for protein quantification. Samples were incubated with DEVD-pNA substrate for 2 h. Colorimetric analysis was performed at 450 nm using the LT-4000 microplate reader (Euroclone, #EMLT4000MS).

### cAMP assay

NSCs were seeded in 6-well plates (50 × 10^5^ cells/well) in NM. After 24 h the medium was replaced with control medium for 2 h, followed by fresh medium with or without GHRH (5 μM) for the indicated times. Cells treated with 50 μM Forskolin for 2 min served as a positive control. 1 × 10^7^ cells were resuspended in Cell Lysis Buffer for 10 min at 4 °C and centrifuged to collect the cytosolic extracts for protein quantification. cAMP was quantified using the Cyclic AMP Assay (R&D Systems, #KGE012B), following the manufacturer’s protocol.

### Western blot

Proteins (30-50 μg) from cell and tissue lysates were resolved in 10% SDS-PAGE (12% for Bcl-2, BAX and CDK5) and transferred onto a nitrocellulose membrane as described [15, 36, 46]. After blocking with 5% BSA in PBS with 0.1% Tween for 1 h (Sigma-Aldrich, #P1379-500ML) for 1 h at room temperature, membranes were incubated overnight at 4 °C with primary antibodies (Supplementary Table 1). Antibody dilutions: 1:500 for CDK5 and synaptophysin, 1:1000 for GHRH, GHRH-R, P-CREB, P-Akt, P-ERK, P-GSK-3β, BAX, P-Tau, P-NF-κB p65, and BACE1, and 1:10000 for NeuN and GFAP. Blots were reprobed with actin antibody (1:500) or corresponding total antibodies for normalization (1:500 for Tau and 1:1000 for CREB, Akt, ERK, GSK-3β, and NF-κB p65). Immunoreactive proteins were detected using horseradish peroxidase-conjugated secondary antibodies (Supplementary Table 2): mouse anti-goat (1:4000), goat anti-mouse, or goat anti-rabbit (1:10000), followed by enhanced chemiluminescence (ECL) detection with a ChemiDoc XRS system (Bio-Rad). Densitometric analysis was performed using Quantity One software (Bio-Rad). The full-length, uncropped original Western blots are provided in the Supplementary Materials.

### RT-PCR and real-time PCR

Total RNA was isolated from NSCs, differentiated SH-SY5Y, and cerebral tissues, then reverse-transcribed into cDNA (1 μg RNA), as described [69]. Real-time PCR was conducted using 50 ng cDNA, 100 nM of each primer, and the Luna Universal qPCR Master Mix (New England BioLabs, # M3003E) on the ABI-Prism 7300 system (Applied Biosystems). Primer sequences were designed using Primer 3 Software (http://www.primer3.org/, RRID: SCR_003139), (Supplementary Table 3). 18S rRNA served as endogenous control, and relative quantification was performed using the comparative 2^−ΔΔCt method.

### Acetylcholinesterase activity assay

SH-SY5Y cells were seeded in 6-well plates in NM (5 × 10⁵ cells per well), then differentiated for 10 days. After differentiation, the medium was replaced with control medium for 6 h, followed by fresh medium with or without GHRH (5 µM) or Aβ_1-42_ (10 µM) for 24 h. Briefly, cells were resuspended in PBS, sonicated and centrifuged to collect cytosolic extracts for protein quantification. Acetylcholinesterase (AChE) activity was measured by Acetylcholinesterase Activity Assay Kit (Sigma-Aldrich, #CS0003). Samples (30 μg) were incubated with Substrate mix for 5 min, and absorbance was recorded at 412 nm using LT-4000 microplate reader (Euroclone, #EMLT4000MS).

### Animals and ethical statement

GHRH-R agonist MR-409 [N-Me-Tyr1, D-Ala2, Orn12, Abu15, Orn21, Nle27, Asp28)-hGHRH(1–29)NH-CH3)] was synthesized and purified in the laboratory of A.V.S [23]. The 5xFAD mouse model, expressing five mutations in human APP and PS1 [B6SJL-Tg(APPSwFlLon, PSEN1*M146L*L286V)6799Vas/Mmjax] under the Thy1 promoter, was purchased from The Jackson Laboratory. Hemizygous 5xFAD mice were bred at the Animal Facility of the Neuroscience Institute Cavalieri Ottolenghi (NICO) (Turin, Italy) with C57BL6/J mice to maintain both the 5xFAD and non-transgenic wild type (WT) colonies. Genotyping of 5xFAD mice was performed by PCR on DNA extracted from tail clippings, using the following primers: *App* TG, forward 5’-AGGACTGACCACTCGACCAG-3’, *App* TG, reverse 5’-CGGGGGTCTAGTTCTGCAT-3’, *Ps1* TG, forward 5’-AATAGAGAACGGCAGGAGCA-3’, *Ps1* TG, reverse 5’-GCCATGAGGGCACTAATCAT-3’, following the Jackson Laboratory’s protocols. All mice were individually housed in a specific pathogen-free and temperature and humidity-controlled facility with a 12 h light/dark cycle and access to food and water ad libitum. To avoid sex-related differences, only male mice were included in this study. Three-month-old male 5xFAD mice were randomly assigned to two groups (*n* = 14 per group) and treated daily for 90 days as follows: (1) the control group (vehicle, VHL) received subcutaneous injections of 0.1% DMSO (Sigma-Aldrich, #D4540) in a 10% aqueous propylene glycol solution, and (2) the MR-409 group received subcutaneous injections of 20 μg MR-409. A total of 28 mice were used. Sample size was determined using the resource equation approach, ensuring an E value between 10 and 20 to balance statistical power and ethical use of animals [70]. No specific strategy was employed to control potential confounding variables in this study. Efforts were made to minimize the number of animals and reduce their suffering. All mice survived until the completion of drug administration. All experiments were conducted in strict accordance with institutional guidelines and complied with national (D.L. No. 26, 04/03/2014) and international (Directive 2010/63/EU) regulations. The study was approved by the Italian Ministry of Health (protocol #1061/2024-PR) and authorized by the Animal Care Committee of the University of Turin.

### Tissue preparation

Mice were anesthetized via intraperitoneal injection of a ketamine/xylazine solution (80 mg/kg ketamine, 10 mg/kg xylazine), and tissues were collected for analysis. For real-time PCR and Western blotting, fresh brain tissues were isolated post-decapitation, rapidly frozen in liquid nitrogen, and stored at –80 °C. For immunofluorescence, mice were perfused transcardially with 0.9% saline followed by 4% paraformaldehyde (PFA, Sigma-Aldrich, #158127). Brains were post-fixed in 4% PFA, incubated in 30% sucrose at 4 °C, embedded in cryostat medium, and sectioned into 40 μm thick slices.

### Immunofluorescence analysis

Immunofluorescence analysis on cultured cells and brain tissues was performed as described [71, 72]. Fixed cells were permeabilized with 0.1% Triton X-100 (Sigma-Aldrich, #X100) for 5 min, blocked with normal goat serum (1:10, Jackson Immunoresearch, #005-000-121) and incubated overnight with primary antibodies against NeuN, GFAP, Nestin (1:250), and β-catenin (1:150), followed by Alexa Fluor 488-conjugated secondary antibodies (1:250) and DAPI nuclear staining (1:1000, Sigma-Aldrich #D9542) (Supplementary Table 1 and 2). Images were acquired using a Leica DM2000 fluorescence microscope equipped with a DFC340 FX camera. Brain sections (40 μm-thick) were incubated overnight with primary antibodies: 6E10 (1:2000), AT8 (1:200), Iba1 (1:1000), NeuN (1:250), and GFAP (1:3000) diluted in PBS containing 2% Triton X-100 and 1.5% normal donkey serum (Jackson ImmunoResearch, #017-000-121). Then, slices were incubated with secondary antibodies (Cy3-conjugated donkey anti-mouse or anti-goat IgG, Alexa Fluor 467-conjugated donkey anti-rabbit, and Alexa Fluor 488-conjugated donkey anti-mouse IgG) (all at 1:400), and counterstained with DAPI (1:1000) (Supplementary Table 1 and 2). Images were acquired using Zeiss Axioscan Z.1 system (Zeiss) at 10x (Plan-Apochromat, 10x/0.45 M27) and 20x (Plan-Apochromat, 20x/0.8 M27) magnification, and acquisition was performed with Leica TCS SP5 confocal laser scanning microscope (DM6000CS, Leica) with a 40x/1.30 oil objective.

### Image analysis

To analyze amyloid plaque deposition and astrogliosis, brain sections were imaged at 10x magnification (Plan-Apochromat 10x/0.45 M27) using the Zeiss Axioscan Z.1 system. Slice alignment and registration were performed with the ABBA semi-automated workflow, a Fiji plugin, using the Allen Mouse Brain Atlas (CCFv3) as described [73]. Annotated slices were then analyzed in QuPath (v0.5.1, RRID:SCR_018257) to compare VHL and MR-409 groups. To assess plaque burden and astrogliosis, 6E10- and GFAP-positive areas were identified using a Pixel Classifier. The proportion of immunopositive pixels (μm²) within the total region of interest (ROI) area (μm²) was then calculated. Amyloid plaques were manually counted and converted into QuPath Detections, then analyzed to measure their area (μm²) using the same classifier. Tau phosphorylation at serine 202 and threonine 205 (AT8) was assessed in the hippocampal regions CA1, CA3, and DG using a Leica TCS SP5 confocal microscope (DM6000CS) at 40x magnification. Four images were captured per subregion from three slices per sample (144 images per group). The AT8-positive area was quantified in Fiji using the Area Fraction measurement. For microglial analysis, Iba1-stained sections were imaged at 20x and 40x magnification with a Zeiss Axioscan microscope and an ApoTome Fluorescence Microscope at 40x magnification (EC Plan-Neofluar 40x/0.75 M27), respectively. ROIs, cortex, hippocampus, and thalamus were defined in Fiji based on the Allen Mouse Brain Atlas. Iba1-positive cell density was calculated per unit area within each ROI. Microglial activation was analyzed in 144 cells per group (excluding those associated with amyloid plaques). Morphological parameters, including the number of branches, maximum branch length, and number of endpoints, were quantified using the Fiji MicrogliaMorphology plugin, as described [74]. Neuronal activity and NeuN expression were assessed by imaging brain sections at 20× magnification using a Zeiss Axioscan microscope. Sections were aligned to the Mouse Brain Atlas as previously described, and NeuN-positive areas within the regions of interest were quantified using Fiji.

### GH and IGF1 analysis

At sacrifice, whole blood was collected and plasma isolated by centrifugation at 2000 × g for 15 min at 4 °C. GH and IGF1 levels were quantified using a Mouse GH ELISA Kit (MyBiosource, #MBS2700017) and IGF1 (Abcam, #ab100695) ELISA Kits, following the manufacturers’ protocols.

### Behavioral tests

#### Novel object recognition (NOR)

The NOR test evaluates short-term memory based on the spontaneous preference of rodents for novel objects [75]. Mice were placed in the testing room 1 h prior to the experiment and kept in complete darkness to minimize stress and visual cues. The apparatus consisted of an opaque Plexiglas chamber (50 × 25 × 25 cm). Mice (*n* = 6 per group) were acclimated to the arena for 5 min before testing. During the training phase, each mouse was allowed to explore the arena containing two identical objects for 10 min. After a 60-min retention interval, the testing phase was conducted by placing the animals in the same apparatus containing one familiar object and one novel object that differed in shape. Mice were allowed to explore freely for 10 min. Both phases were performed under dark conditions, and behavior was recorded with an infrared camera positioned above the apparatus. The first 5 min of the test phase were analyzed using EthoVision XT video tracking software (Noldus, Wageningen, The Netherlands) to quantify object exploration. The discrimination index (DI), i.e., the time (T) spent exploring the novel object (N) relative to the total amount of exploration of the novel (N) and familiar objects (F): [DI = (TN − TF)/(TN + TF)]. Q The DI ranges from -1 to +1, with positive values indicating more time spent with the novel object and negative values indicating more time spent with the familiar object.

#### Y-maze

The Y-maze test was conducted to evaluate spatial memory. The test consisted of the spontaneous exploration of a Y-shaped maze with three identical plastic arms positioned at 120° angles from each other. Prior to testing, mice were placed in the experimental room for 1 h and kept in complete darkness to reduce environmental stress.

During the habituation phase, each mouse (*n* = 6 per group) was placed in the maze with one arm blocked (novel arm), allowing free exploration of the two open arms for 5 min. After a 1-h retention interval, the novel arm was opened, and the mouse was returned to the start arm and allowed to explore freely for another 10 min. In this phase, mice are expected to spend more time exploring the novel arm, with fewer entries into the previously visited arms. Exploratory behavior during the initial 5 min of the test was recorded and analyzed using EthoVision XT software, including exploration time and discrimination index.

### Statistical analysis

Data are presented as mean ± SEM. Statistical analysis was performed using a two-tailed Student’s *t* test with Welch’s correction or one-way ANOVA followed by Dunnett’s or Tukey’s post hoc tests, as appropriate. All analysis were conducted using GraphPad Prism 8.0 (GraphPad Software, RRID: SCR_002798), with significance set at *P* < 0.05.

## Supplementary information

Supplemental

Original Western blots

## Data Availability

All the data supporting the findings of this study are available within the article and Supplementary Information. Raw data, material or methods used or produced in this study can be shared for research purposes upon reasonable request to the corresponding author.
