# Supplementary material for: Growth hormone-releasing hormone attenuates amyloid deposition and neuroinflammation in Alzheimer’s disease models
Source: Cell Death Dis. 2026 Apr 7;17(1):494. doi: 10.1038/s41419-026-08699-w (PMC13187343; doi:10.1038/s41419-026-08699-w)
Supplement: Supplementary file 1 — Supplemental [file 41419_2026_8699_MOESM1_ESM.pdf]

Supplementary data

Growth hormone-releasing hormone attenuates amyloid deposition and neuroinflammation in Alzheimer's disease models (Manuscript ID: CDDIS-25-3649).

Supplementary Figures

Supplementary Fig. 1: Survival effect of GHRH at 48 h in NSCs
Supplementary Fig. 2: Signaling pathways mediating the survival and proliferative effects of GHRH
Supplementary Fig. 3: Expression of nestin in differentiating NSCs
Supplementary Fig. 4: Inhibitory effect of Aβ on survival of NSCs.
Supplementary Fig. 5: Signaling pathways involved in the protective effects of GHRH against Aβ-induced toxicity
Supplementary Fig. 6: Effect of GHRH on differentiation of SH-SY5Y cells
Supplementary Fig. 7: Inhibitory effect of Aβ on viability of SH-SY5Y cells
Supplementary Fig. 8: Aβ deposition in 5xFAD mice treated with vehicle (VHL) or MR-409
Supplementary Fig. 9: GFAP immunoreactivity in 5xFAD mice treated with vehicle (VHL) or MR-409
Supplementary Fig. 10: GH and IGF-1 levels in 5xFAD mice

Supplementary Tables

Supplementary Table 1: Primary antibodies used for immunofluorescence (IF) and Western blot (WB) assays
Supplementary Table 2: Secondary antibodies used for immunofluorescence (IF) and Western blot (WB) assays
Supplementary Table 3: Primer sequences used for real-time PCR analysis

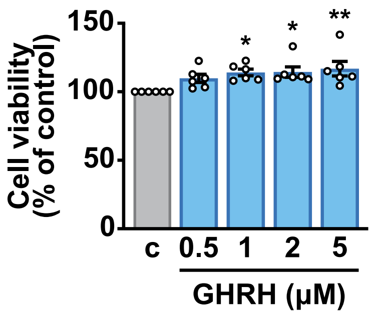

Supplementary Fig. 1: Survival effect of GHRH at 48 h in NSCs. Cell viability assessed by MTT in cells cultured in control medium (c) for 48 h, either alone or in the presence of GHRH, at the indicated concentrations. Results are expressed as percentage of control and are means ± SEM. *$P < 0.05$, **$P < 0.01$ vs. c by one-way ANOVA and Dunnett's post-hoc test ($n = 6$).

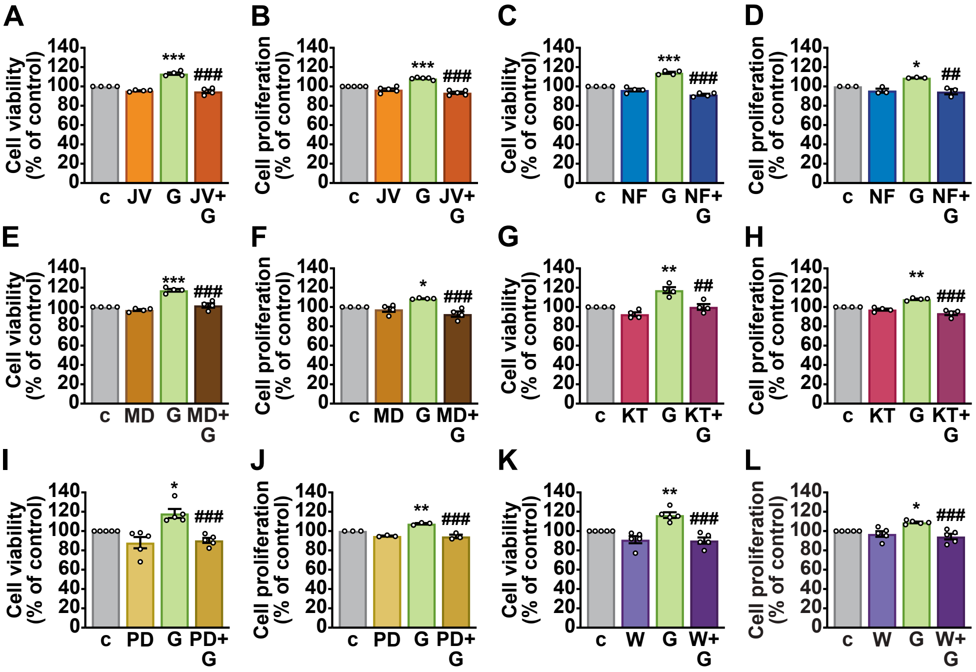

Supplementary Fig. 2: Signaling pathways mediating the survival and proliferative effects of GHRH. NSCs were cultured in growth factor deprived medium (c, control) for 24 h, in either absence or presence of GHRH (5 μM) (G), JV-1-36 (1 nM) (JV) **(A, B)**, NF449 (25 μM) (NF) **(C, D)**, MDL-12330A (100 nM) (MD) **(E, F)**, KT5720 (100 nM) (KT) **(G, H)**, PD98059 (10 μM) (PD) **(I, J)**, or wortmannin (25 nM) (W) **(K, L)**. Cell viability and proliferation were assessed by MTT and BrdU assays, respectively. Results, expressed as percentage of control, are means ± SEM. $*P < 0.05$, $**P < 0.01$, $***P < 0.001$ vs. c; $^{\#\#}P < 0.01$, $^{\#\#\#}P < 0.001$ vs. GHRH by one-way ANOVA and Tukey's post-hoc test (n = 3 for **D** and **J**; n = 4 for **A, C** and **E-H**; n = 5 for **B**, **I** and **K-L**).

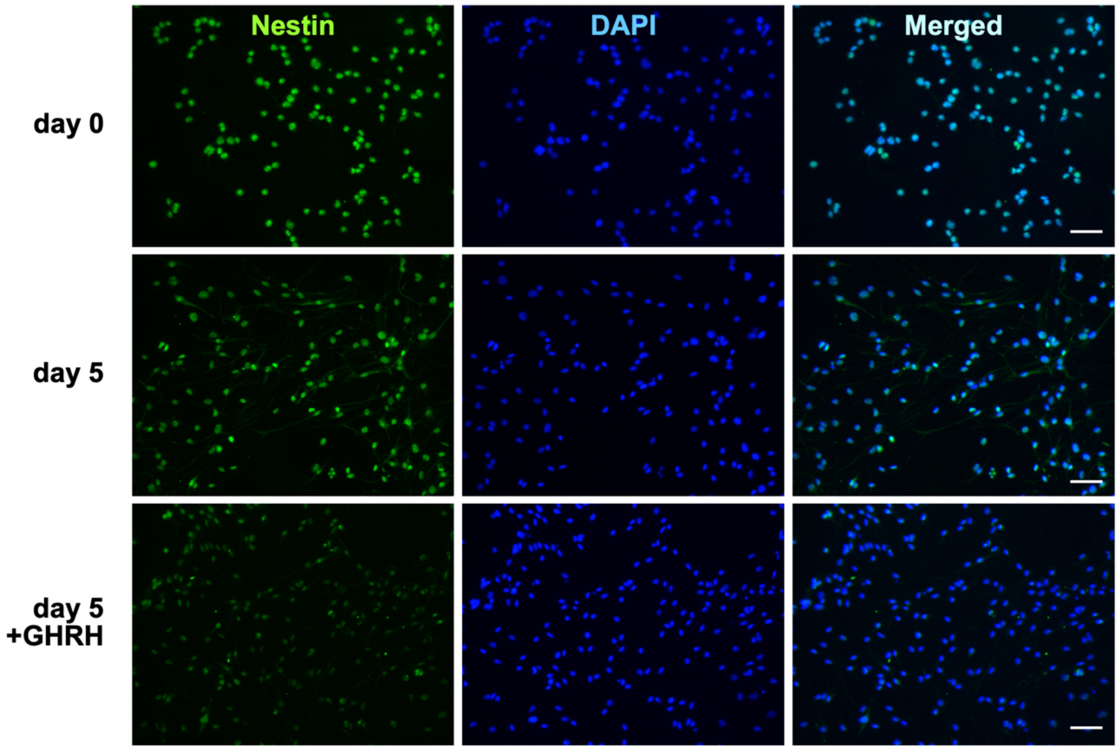

Supplementary Fig. 3: Nestin expression in differentiating NSCs. Representative fluorescence micrographs of cells cultured in normal medium (day 0) and in differentiation medium for 5 days, in either absence or presence of GHRH (5 µM). Cells were stained for nestin (green), nuclei were counterstained with DAPI (blue) (scale bar: 50 µm).

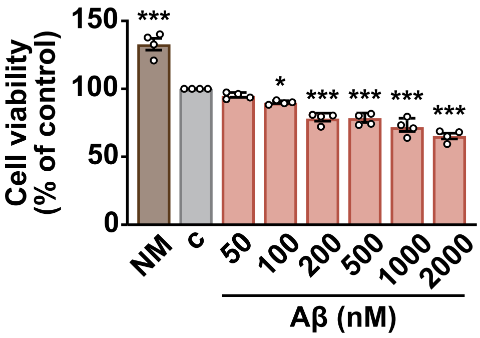

Supplementary Fig. 4: Inhibitory effect of Aβ on survival of NSCs. Cell viability, assessed by MTT, in cells cultured in normal medium (NM) alone or in growth factor deprived medium (c, control) for 24 h without or with $A\beta_{1\text{-}42}$, at the indicated concentrations. Results, expressed as percentage of control, are means ± SEM. *$P < 0.05$, ***$P < 0.001$ vs. c by one-way ANOVA and Dunnett's post-hoc test ($n = 4$).

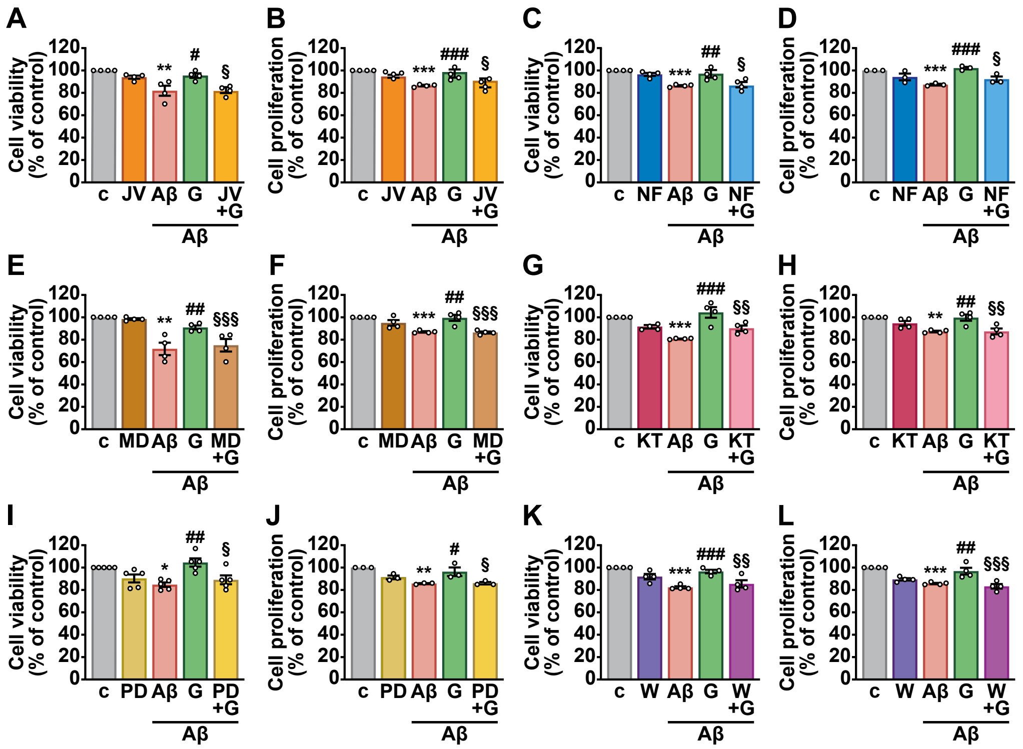

Supplementary Fig. 5: Signaling pathways involved in the protective effects of GHRH against Aβ-induced toxicity. NSCs were cultured in growth factor-deprived medium (c) for 24 h in the absence or presence of $A\beta_{1-42}$ (200 nM) and GHRH (G) (5 μM), and with either JV-1-36 (JV) (1 nM) **(A, B)**, NF449 (NF) (25 μM) **(C, D)**, MDL-12330A (MDL) (100 nM) **(E, F)**, KT5720 (KT) (100 nM) **(G, H)**, PD98059 (PD) (10 μM) **(I, J)**, or wortmannin (W) (25 nM) **(K, L)**. Cell viability and proliferation were assessed by MTT and BrdU assays, respectively. Results, expressed as percentage of control, are means ± SEM. $^{*}P < 0.05$, $^{**}P < 0.01$, $^{***}P < 0.001$ vs. c; $^{\#}P < 0.05$, $^{\#\#}P < 0.01$, $^{\#\#\#}P < 0.001$ vs. Aβ; $^{\S}P < 0.05$, $^{\S\S}P < 0.01$, $^{\S\S\S}P < 0.001$ vs. G+Aβ by one-way ANOVA and Tukey's post-hoc test (n = 3 for d and j; n = 4 for a, c, e-h and k-l; n = 5 for b and i).

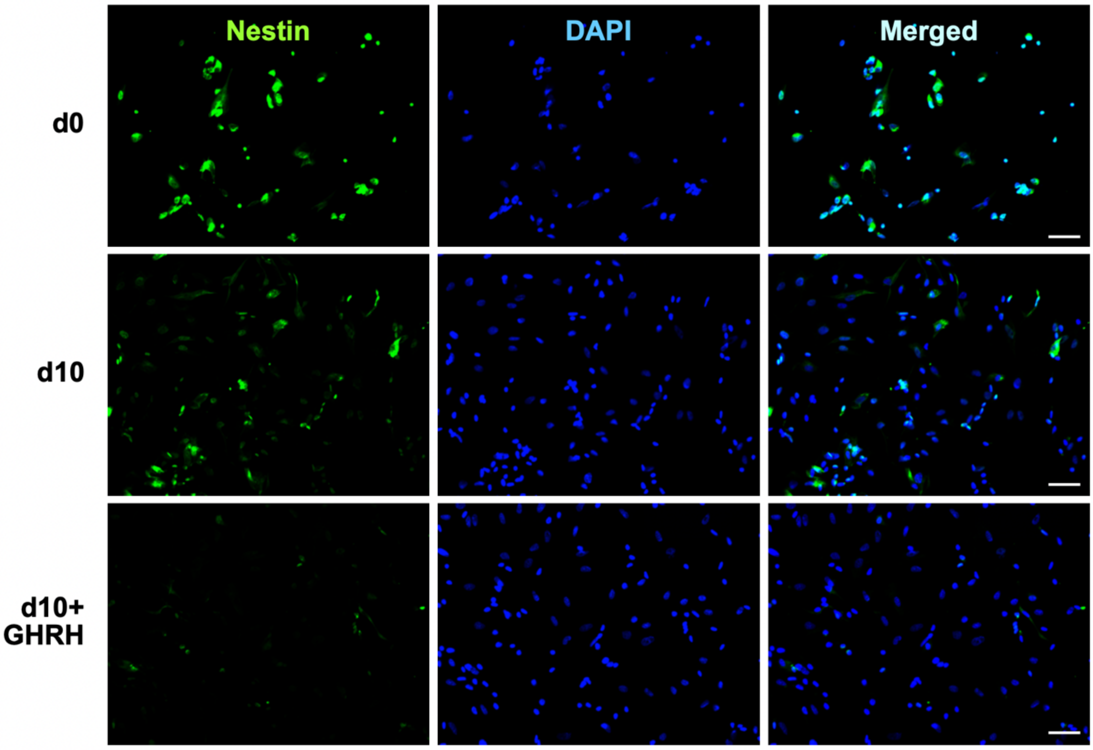

Supplementary Fig. 6: Effect of GHRH on differentiation of SH-SY5Y cells. Representative fluorescence micrographs of cells cultured in normal medium (d, day 0) and in differentiation medium for 10 days, in the absence or presence of GHRH (5 μM). Cells were stained for nestin (green), nuclei were counterstained with DAPI (blue), (scale bar: 50 μm).

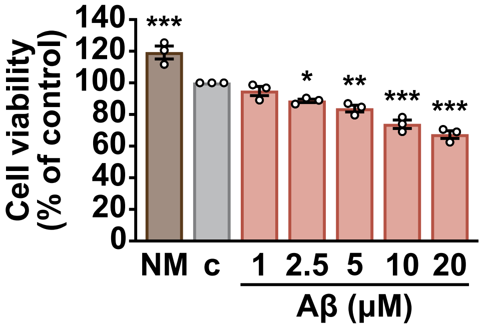

Supplementary Fig. 7: Inhibitory effect of Aβ on viability of SH-SY5Y cells. Cell viability was assessed by MTT in cells cultured in normal medium (NM) or in control medium (c) for 24 h, without or with Aβ, at the indicated concentrations. Results, expressed as percentage of control, are means ± SEM. *$P < 0.05$, **$P < 0.01$, ***$P < 0.001$ vs. c by one-way ANOVA and Dunnett's post-hoc test ($n = 3$).

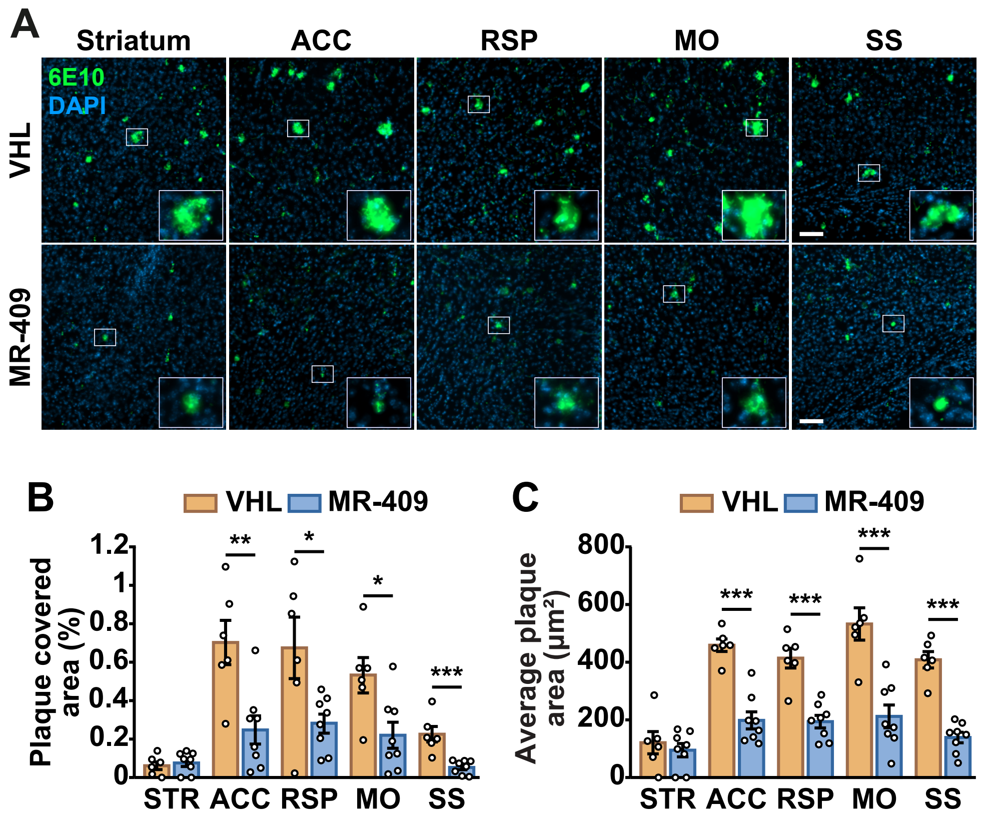

Supplementary Fig. 8: Aβ deposition in 5xFAD mice treated with vehicle (VHL) or MR-409. A Representative Aβ immunostaining with 6E10 antibody (green) of the striatum (STR), anterior cingulate cortex (ACC), retrosplenial cortex (RSP), motor cortex (MO) and somatosensory cortex (SS). Nuclei were counter stained with DAPI (blue). Insets show the magnified plaques. Scale bar: 50 µm Quantification of plaque covered area **(B)** and average plaque area **(C)**. Results are means ± SEM. $^{*}P < 0.05$, $^{**}P < 0.01$, $^{***}P < 0.001$ vs. VHL by unpaired Student's t test. (VHL, $n = 6$; MR-409, $n = 8$).

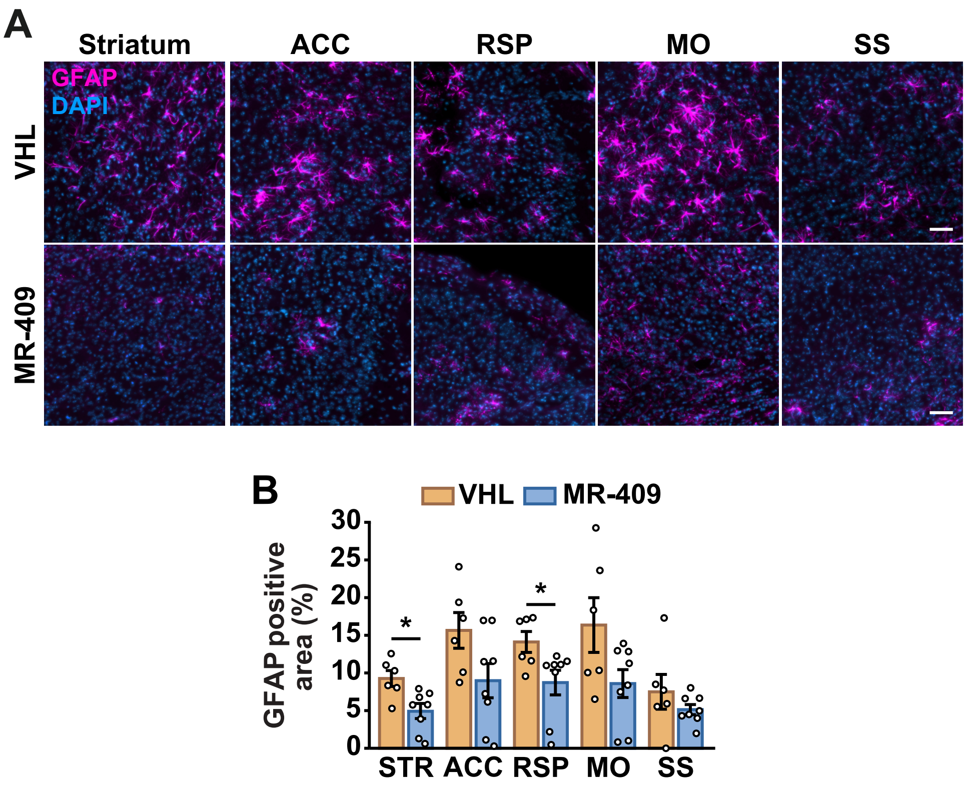

Supplementary Fig. 9: GFAP immunoreactivity in 5xFAD mice treated with vehicle (VHL) or MR-409. **A** Representative GFAP immunostaining (magenta) of the striatum (STR), anterior cingulate cortex (ACC), retrosplenial cortex (RSP), motor cortex (MO) and somatosensory cortex (SS). Nuclei were counter stained with DAPI (blue). **B** Quantification of GFAP positive area in the indicated brain regions. Scale bar: 50 µm. Results are means ± SEM. *$P < 0.05$ vs. VHL by unpaired Student's t test. (VHL, $n = 6$; MR-409, $n = 8$).

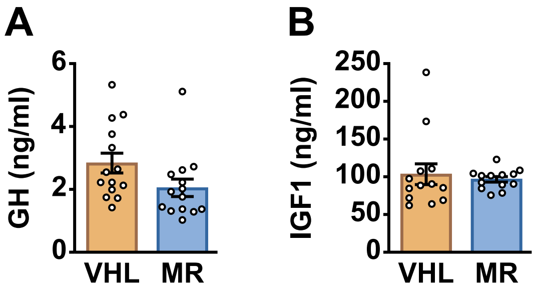

Supplementary Fig. 10: GH and IGF1 levels in 5xFAD mice. Plasma levels of GH **(A)** and IGF1 **(B)** determined by ELISA in mice treated with either vehicle (VHL) or MR-409 (MR). Results are means ± SEM (n = 14).

Supplementary Table 1: Primary antibodies used for immunofluorescence (IF) and Western blot (WB) assays.

Primary antibody	Dilution	Company	Code	RRID
Anti-GHRH-R	1:1000 (WB)	Abcam	Ab76263	RRID:AB_1566283
Anti-GHRH-R [which also recognizes SV1]	1:1000 (WB)	Abcam	Ab28692	RRID:AB_732729
Anti-GHRH	1:1000 (WB)	Abcam	Ab187512	RRID:AB_3678595
Anti-Actin	1:500 (WB)	Santa Cruz Biotechnology	Sc-376421	RRID:AB_11149557
Anti-P-CREB (Ser133)	1:1000 (WB)	Cell signaling Technology	9198S	RRID:AB_2561044
Anti-CREB	1:1000 (WB)	Cell signaling Technology	9197S	RRID:AB_331277
Anti-P-ERK1/2 (Thr202/Tyr204)	1:1000 (WB)	Cell signaling Technology	9101S	RRID:AB_331646
Anti-ERK1/2	1:1000 (WB)	Santa Cruz Biotechnology	Sc-292838	RRID:AB_2650548
Anti-P-Akt (Ser473)	1:1000 (WB)	Cell signaling Technology	9271S	RRID:AB_329825
Anti-Akt	1:1000 (WB)	Cell signaling Technology	4685S	RRID:AB_2225340
Anti-P-GSK-3β (Ser9)	1:1000 (WB)	Cell signaling Technology	9336S	RRID:AB_331405
Anti-GSK-3β	1:1000 (WB)	Cell signaling Technology	9315S	RRID:AB_490890
Anti-NeuN	1:10000 (WB)<br>1:250 (IF)	Abcam	Ab177487	RRID:AB_2532109
Anti-GFAP	1:10000 (WB)<br>1:250 (IF on cultured cells)	Abcam	Ab7260	RRID:AB_305808
Anti-GFAP	1:3000 (IF on brain tissue)	Abcam	Ab53554	RRID:AB_880202
Anti-Nestin	1:250 (IF)	Abcam	Ab92391	RRID:AB_10561437
Anti-BAX	1:1000 (WB)	Cell signaling Technology	5023S	RRID:AB_10557411
Anti-Bcl-2	1:500 (WB)	Santa Cruz Biotechnology	Sc-7382	RRID:AB_626736
Anti-P-Tau (Ser396)	1:1000 (WB)	Santa Cruz Biotechnology	Sc-12414	RRID:AB_656749
Anti-P-Tau (Ser202, Thr205) (AT8)	1:200 (IF)	Invitrogen	MN1020	RRID:AB_223647
Anti-Tau (Tau-5)	1:500 (WB)	Invitrogen	AHB0042	RRID:AB_2536235
Anti-β-catenin	1:150 (IF)	Cell signaling Technology	8480	RRID:AB_11127855
Anti-P-NFκB p65 (Ser536)	1:1000 (WB)	Cell signaling Technology	3033S	RRID:AB_331284
Anti-NFκB p65	1:1000 (WB)	Cell signaling Technology	8242S	RRID:AB_10859369
Anti-Amyloid-β (6E10 clone)	1:2000 (IF)	BioLegend	803001	RRID:AB_2564653
Anti-BACE1	1:1000 (WB)	Abcam	Ab263901	RRID:AB_3696796
Anti-CDK5	1:500	Santa Cruz Biotechnology	Sc-249	RRID:AB_627240
Anti-Iba1	1:1000 (IF)	FujiFilm Wako Chemicals	019-19741	RRID:AB_839504
Anti-Synaptophisin	1:500	Abcam	Ab8049	RRID:AB_2198854

Supplementary Table 2: Secondary antibodies used in immunofluorescence (IF) and Western blot (WB) assays.

Secondary antibody	Dilution	Company	Code	RRID
Peroxidase AffiniPure Goat Anti-Rabbit IgG (H+L)	1:10000	Jackson ImmunoResearch	111-035-144	RRID:AB_2307391
Peroxidase AffiniPure Goat Anti-Mouse IgG (H+L)	1:10000	Jackson ImmunoResearch	115-035-003	RRID:AB_10015289
Mouse Anti-Goat IgG Fc-HRP	1:4000	SouthernBiotech	6158-05	RRID:AB_2796222
Goat anti-Rabbit IgG (H+L) Secondary Antibody, Alexa Fluor 488	1:250	Invitrogen	A-11034	RRID:AB_2576217
Cy3-AffiniPure Donkey Anti-Mouse IgG (H+L)	1:400	Jackson ImmunoResearch	715-165-151	RRID:AB_2315777
Cy3-AffiniPure Donkey Anti-Goat IgG (H+L)	1:400	Jackson ImmunoResearch	705-165-147	RRID:AB_2307351
Alexa Fluor 647 AffiniPure Donkey Anti-Rabbit IgG (H+L)	1:400	Jackson ImmunoResearch	711-605-152	RRID:AB_2492288
Alexa Fluor 488 AffiniPure Donkey Anti-Mouse IgG (H+L)	1:400	Jackson ImmunoResearch	715-545-151	RRID:AB_2341099

Supplementary Table 3. Primer sequences used for real-time PCR analysis

Target gene	Sequences	
18S (NR_146144.1)	Forw<br>Rev	5'-CCCATTCGAACGTCTGCCCTACT- 3'<br>5'-TGCTGCCTTCCTTGGATGTGGTA- 3'
**Rat**		
Tubb3 (NM_139254.2)	Forw<br>Rev	5'-TAGACCCCAGCGGCAACTAT- 3'<br>5'-GTTCCAGGCTCCAGGTCCACC- 3'
Gfap (NM_017009.2)	Forw<br>Rev	5'-CTCAGTACGAGGCAGTGGCC- 3'<br>5'-CGGGAAGCAACGTCTGTGA- 3'
Ifng (NM_138880.3)	Forw<br>Rev	5'-AGTCTGAAGAACTATTTTAACTCAAGTAGCAT- 3'<br>5'-CTGGCTCTCAAGTATTTTCGTGTTAC- 3'
Il1b (NM_031512.2)	Forw<br>Rev	5'-CACCTCTCAAGCAGAGCACAG- 3'<br>5'-GGGTTCCATGGTGAAGTCAAC- 3'
Il6 (NM_012589.2)	Forw<br>Rev	5'-GCCCTTCAGGAACAGCTATGA- 3'<br>5'-TGTCAACAACATCAGTCCCAAGA- 3'
**Mouse**		
Gfap (NM_001131020.1)	Forw<br>Rev	5'-CTCAGTACGAGGCAGTGGCC- 3'<br>5'-CGGGAAGCAACGTCTGTGA- 3'
Il1b (NM_008361.4)	Forw<br>Rev	5'-CTGCAGCTGGAGAGTGTGGAT- 3'<br>5'-CTCCACTTTGCTCTTGACTTCTATCTT- 3'
Il6 (NM_031168.2)	Forw<br>Rev	5'-TTCCATCCAGTTGCCTTCTTG- 3'<br>5'-TTGGGAGTGGTATCCTCTGTGA- 3'
Il10 (NM_010548.2)	Forw<br>Rev	5'-GCATGGCCCAGAAATCAAGG-3'<br>5'-GAGAAATCGATGACAGCGCC-3'
Tnfa (NM_001278601.1)	Forw<br>Rev	5'-TCTCATCAGTTCTATGGCCC- 3'<br>5'-GGGAGTAGACAAGGTACAAC- 3'
Bdnf (NM_007540.4)	Forw<br>Rev	5'-CCATAAGGACGCGGACTTGTAC- 3'<br>5'-AGACATGTTTGCGGCATCCAGG- 3'
Vgf (NM_001039385.1)	Forw<br>Rev	5'-CTTTGACACCCTTATCCAAGGCG- 3'<br>5'-GCTAATCCTTGCTGAAGCAGGC- 3'
Ngf (NM_013609.3)	Forw<br>Rev	5'-GTTTTGCCAAGGACGCAGCTTTC- 3'<br>5'-GTTCTGCCTGTACGCCGATCAA- 3'
Dlg4 (NM_007864.3)	Forw<br>Rev	5'- TCAACAGTGTGGGGCTAGAG- 3'<br>5'- TGCCCAAGTAGCTGCTATGA-3'
Keap1 (NM_016679.4)	Forw<br>Rev	5'- GCTACAACCCCATGACCAAC-3'<br>5'- GGCTCATATCTCTCCACGCT-3'
Nfe2l2 (NM_010902.5)	Forw<br>Rev	5'- CAGCATAGAGCAGGACATGGAG-3'<br>5'- GAACAGCGGTAGTATCAGCCAG-3'
**Human**		
BDNF (NM_170735.6)	Forw<br>Rev	5'-CATCCGAGGACAAGGTGGCTTG- 3'<br>5'- GCCGAACTTTCTGGTCCTCATC-3'
SYP (NM_003179.3)	Forw<br>Rev	5'-TCGGCTTTGTGAAGGTGCTGCA- 3'<br>5'-TCACTCTCGGTCTTGTTGGCAC- 3'
PSD-95 (NM_001128827.4)	Forw<br>Rev	5'-TCCACTCTGACAGTGAGACCGA- 3'<br>5'-CGTCACTGTCTCGTAGCTCAGA-3'
