# Supplementary material for: Growth hormone-releasing hormone attenuates amyloid deposition and neuroinflammation in Alzheimer’s disease models
Source: Cell Death Dis. 2026 Apr 7;17(1):494. doi: 10.1038/s41419-026-08699-w (PMC13187343; doi:10.1038/s41419-026-08699-w)

Growth hormone-releasing hormone attenuates amyloid deposition and neuroinflammation in Alzheimer's disease models (CDDIS-25-364R)

Francesca Pedrolli et al.

Original Western blots

GHRH-R expression in NSCs cells (Figure 1A)

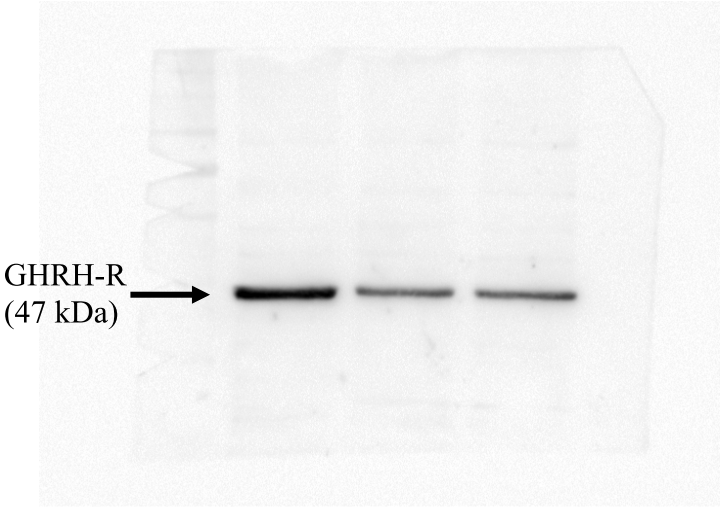

Actin for GHRH-R expression in NSCs cells (Figure 1A)

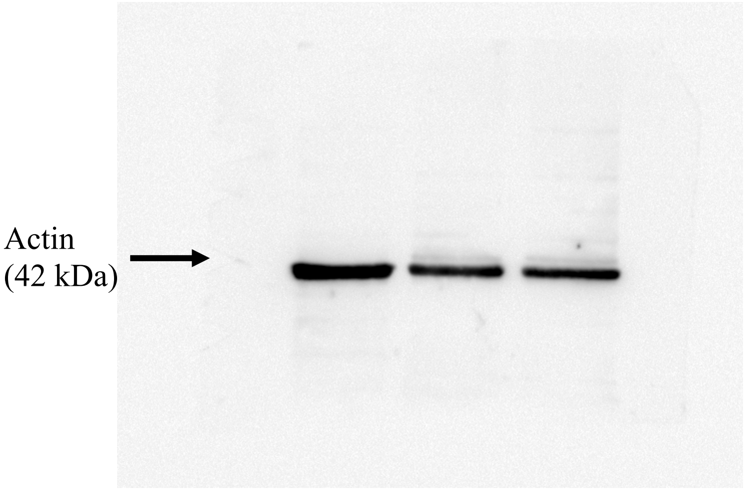

GHRH expression in NSCs cells (Figure 1B)

GHRH
(12 kDa)

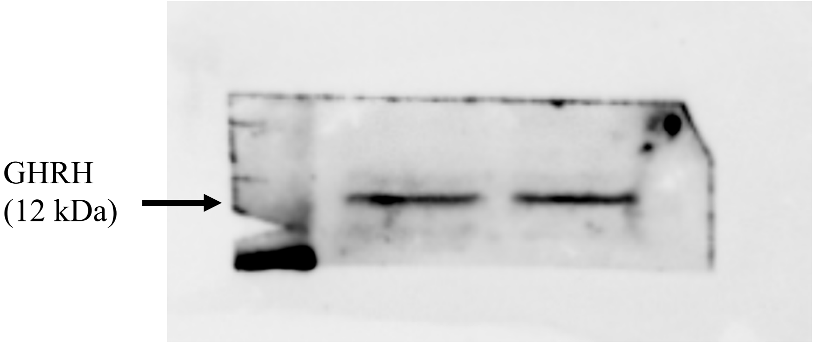

Actin for GHRH expression in NSCs cells (Figure 1B)

Actin
(42 kDa)

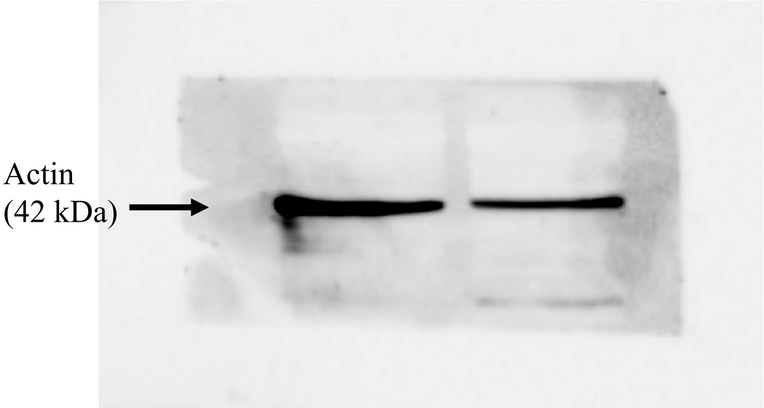

P-CREB in NSCs cells (Figure 1G)

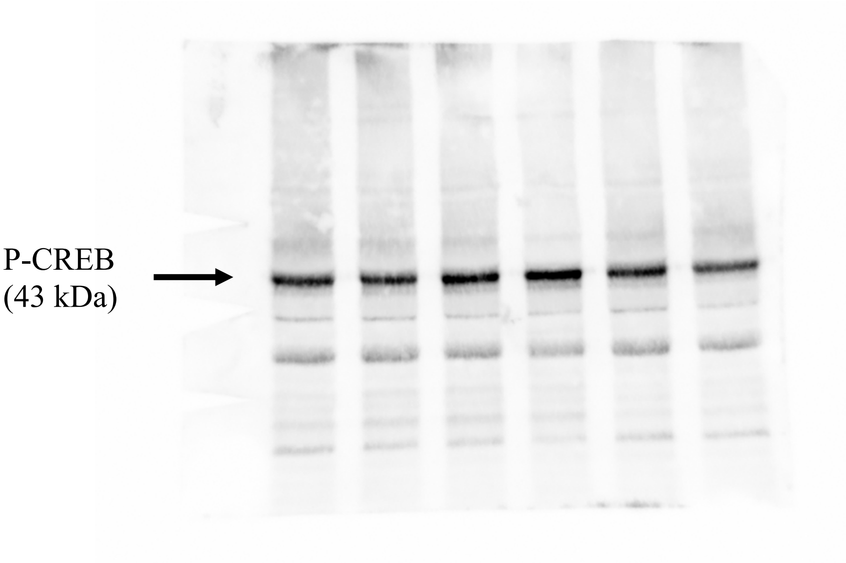

CREB in NSCs cells (Figure 1G)

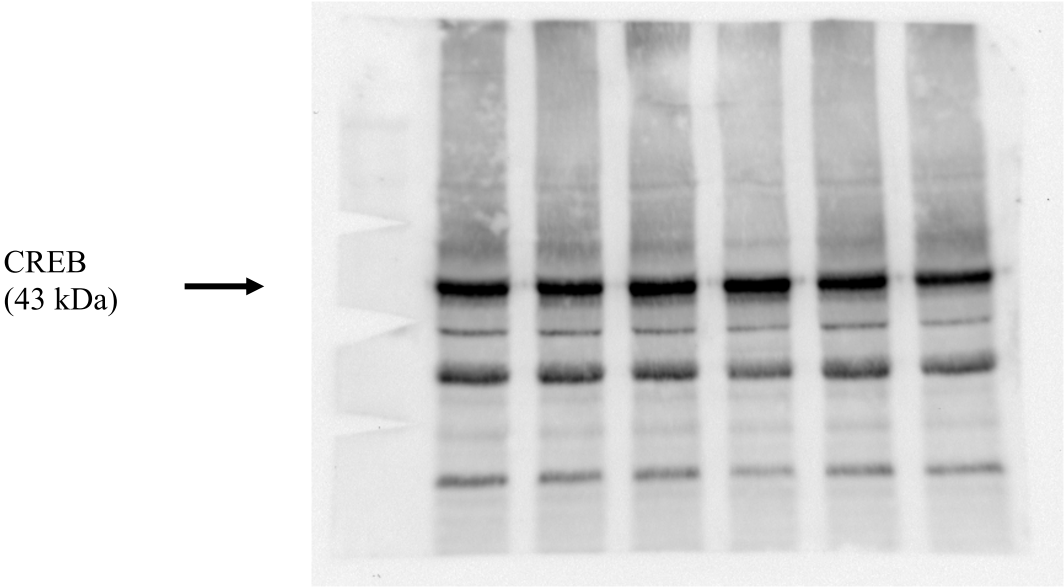

P-ERK1/2 in NSCs cells (Figure 1H)

P-ERK1/2
(42, 44 kDa)

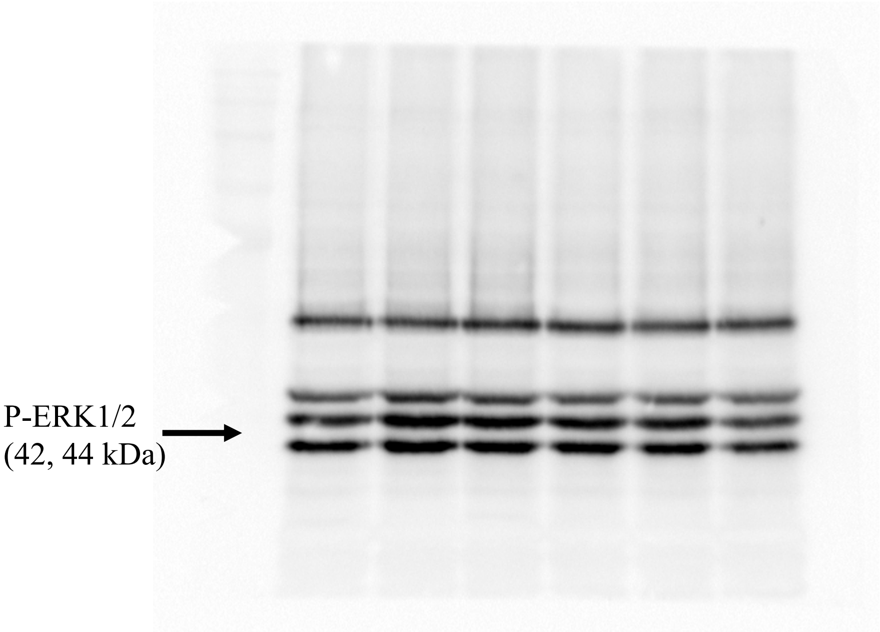

ERK1/2 in NSCs cells (Figure 1H)

ERK1/2
(42, 44 kDa)

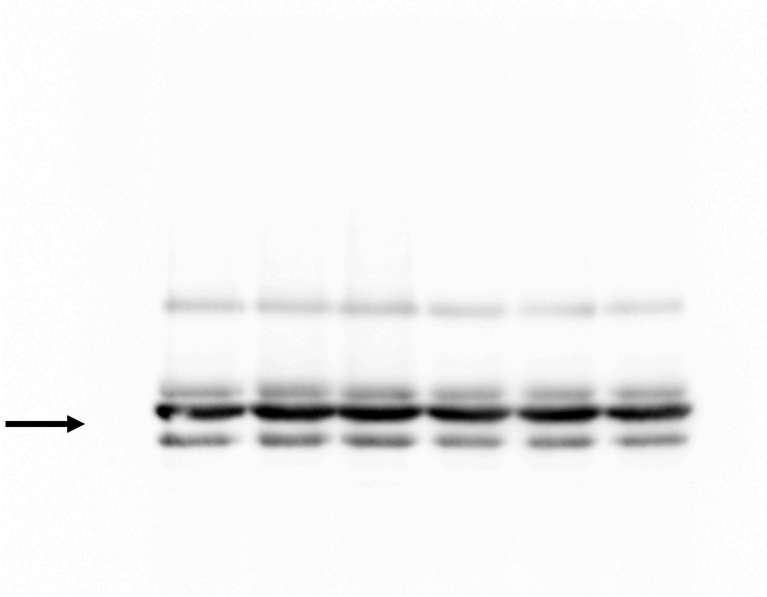

P-Akt in NSCs cells (Figure 1I)

P-Akt
(60 kDa)

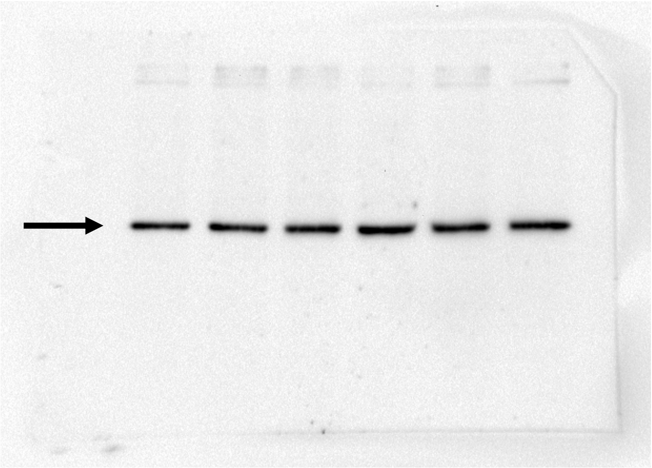

Akt in NSCs cells (Figure 1I)

Akt
(60 kDa)

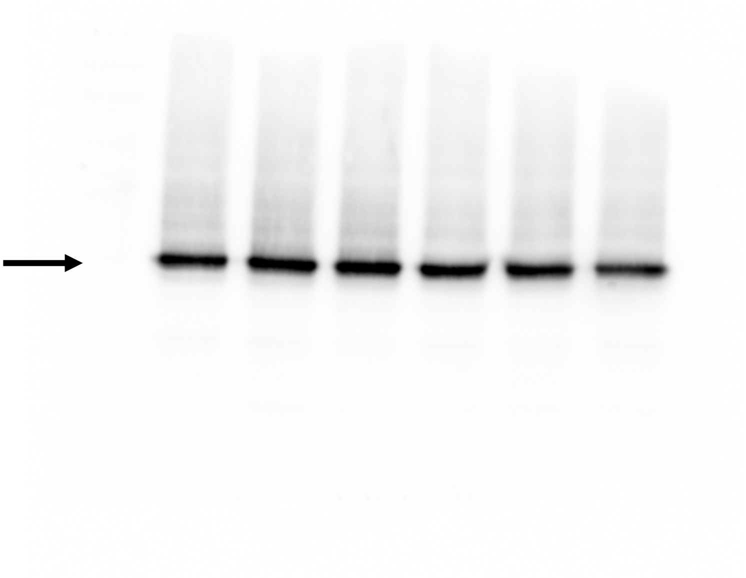

P-GSK-3β in NSCs cells (Figure 1J)

P-GSK-3β
(46 kDa)

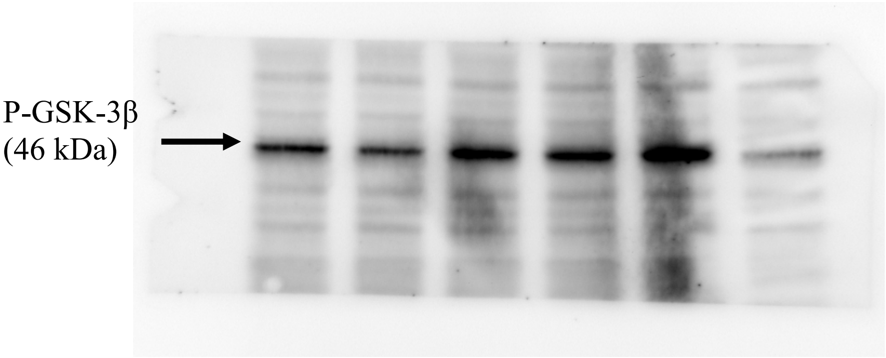

GSK-3β in NSCs cells (Figure 1J)

GSK-3β
(46 kDa)

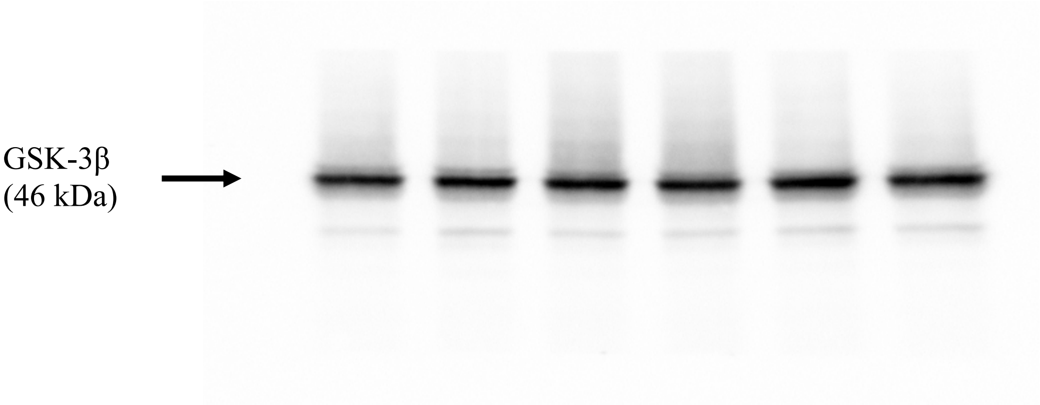

NeuN in NSCs cells (Figure 1L)

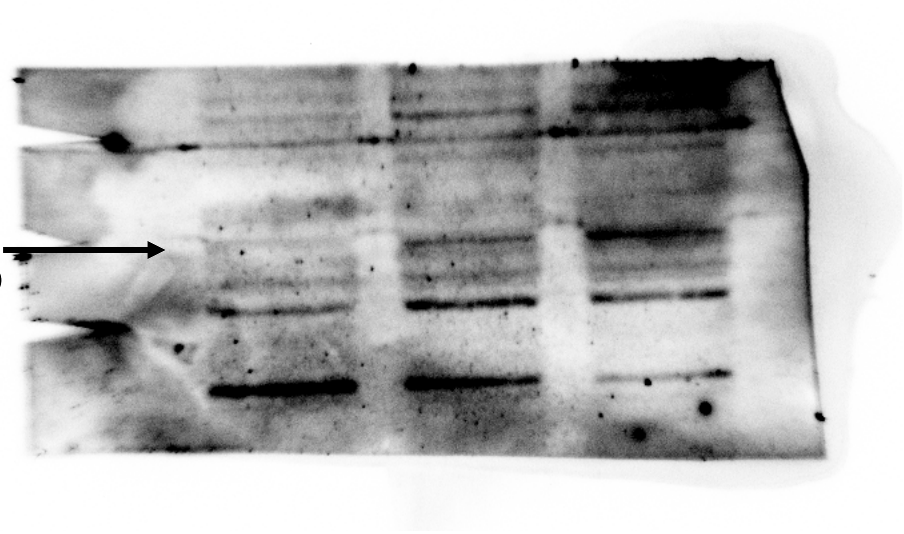

Actin for NeuN in NSCs cells (Figure 1L)

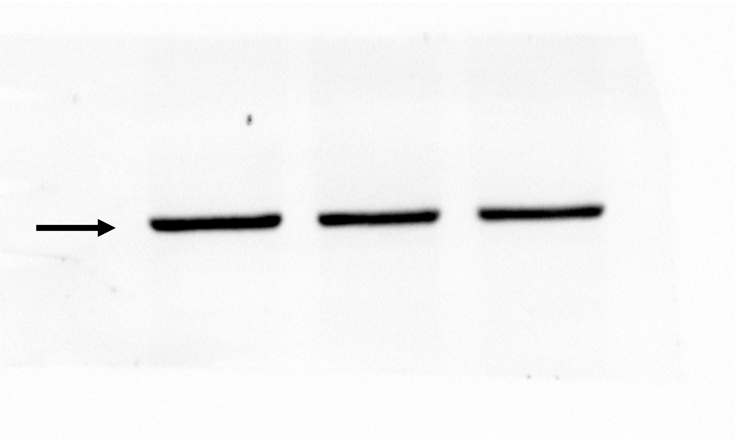

GFAP in NSCs cells (Figure 1M)

GFAP
(50 kDa)

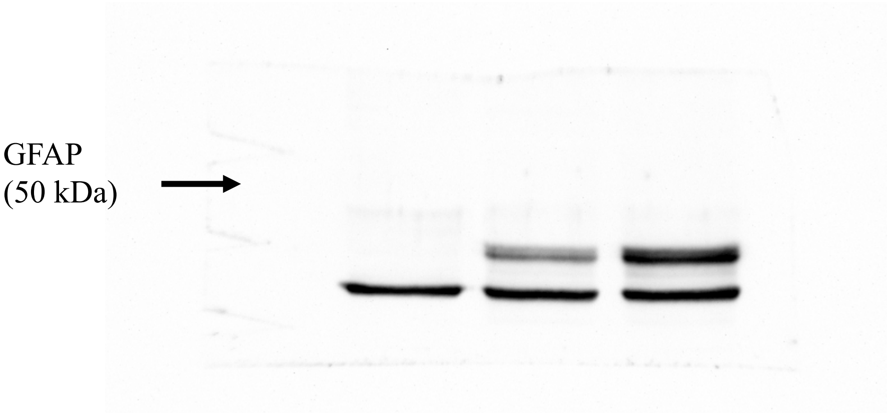

Actin for GFAP in NSCs cells (Figure 1M)

Actin
(42 kDa)

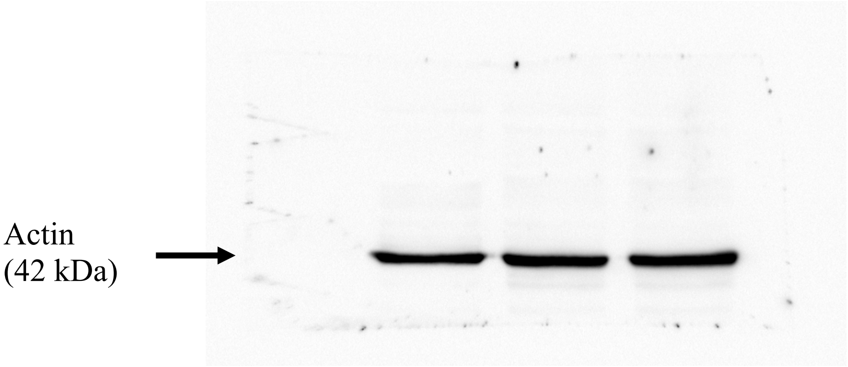

BAX in NSCs cells (Figure 2D)

BAX
(20 kDa)

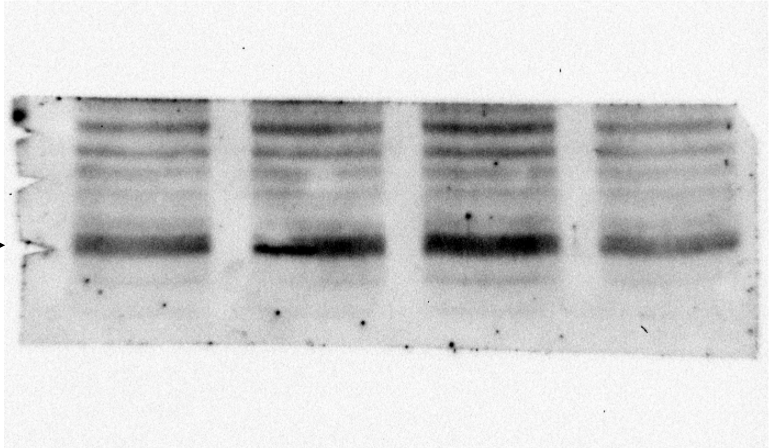

Actin for BAX in NSCs cells (Figure 2D)

Actin
(42 kDa)

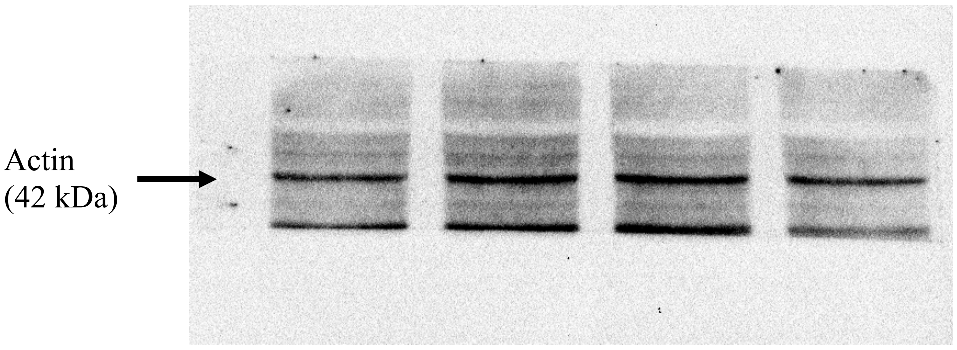

Bcl-2 in NSCs cells (Figure 2E)

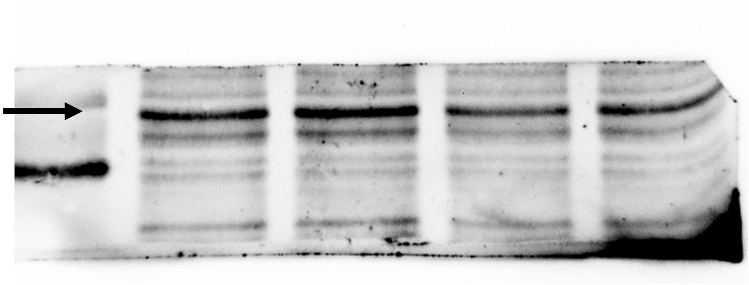

Actin for Bcl-2 in NSCs cells (Figure 2E)

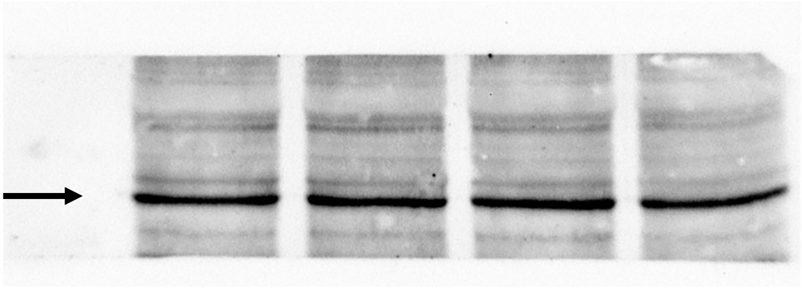

P-CREB in NSCs cells (Figure 2F)

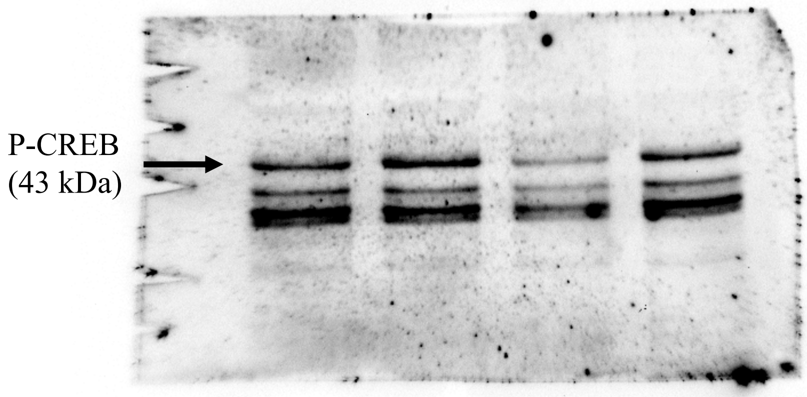

CREB in NSCs cells (Figure 2F)

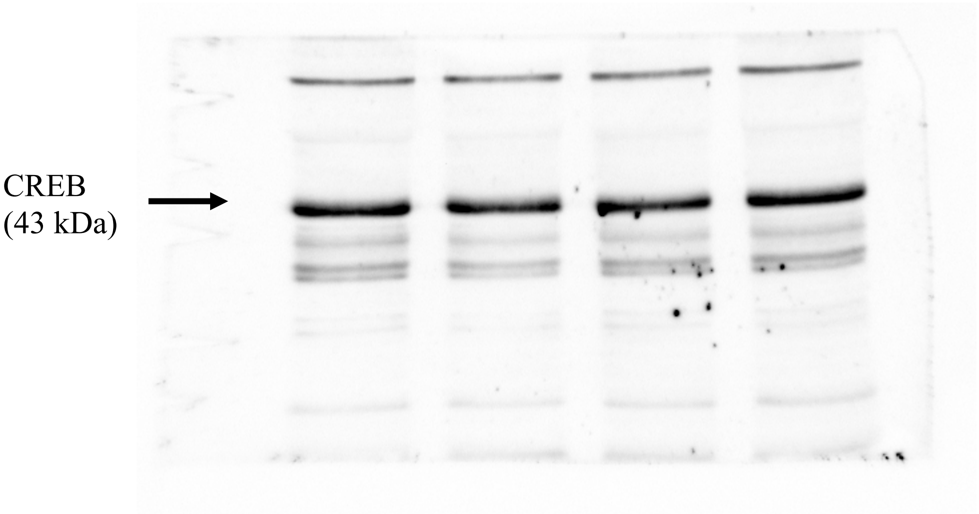

P-ERK1/2 in NSCs cells (Figure 2G)

P-ERK1/2
(42, 44 kDa)

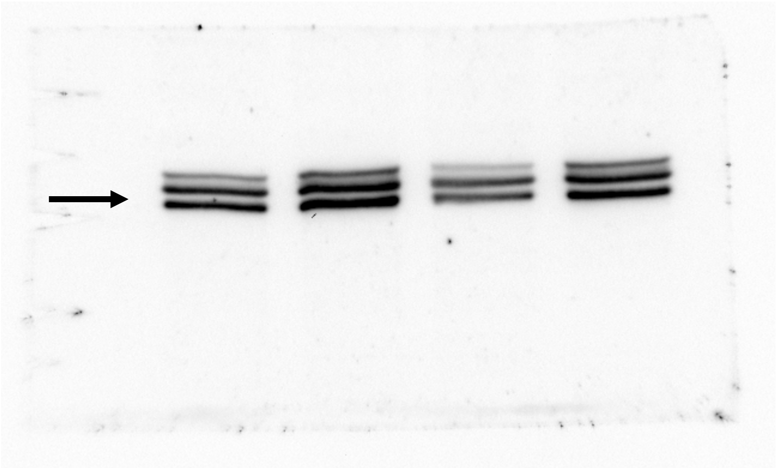

ERK1/2 in NSCs cells (Figure 2G)

ERK1/2
(42, 44 kDa)

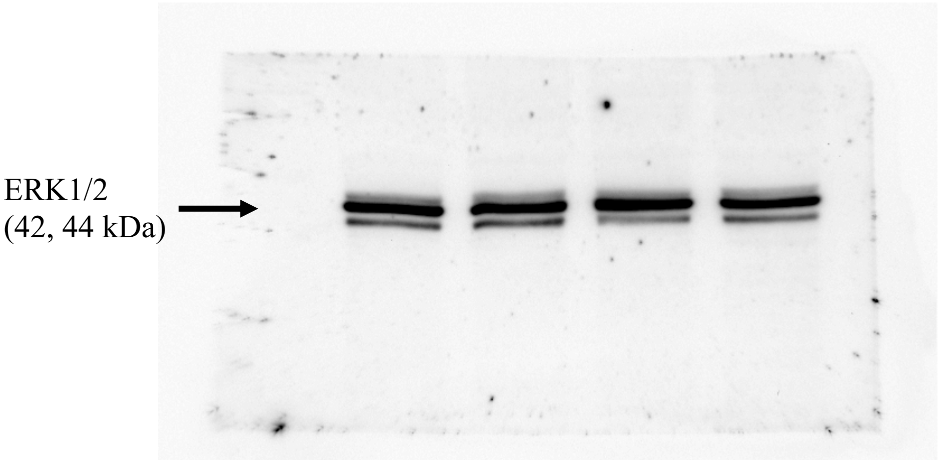

P-Akt in NSCs cells (Figure 2H)

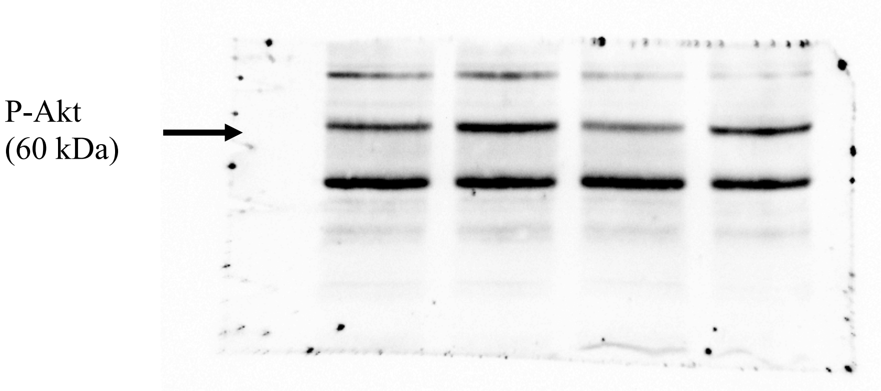

Akt in NSCs cells (Figure 2H)

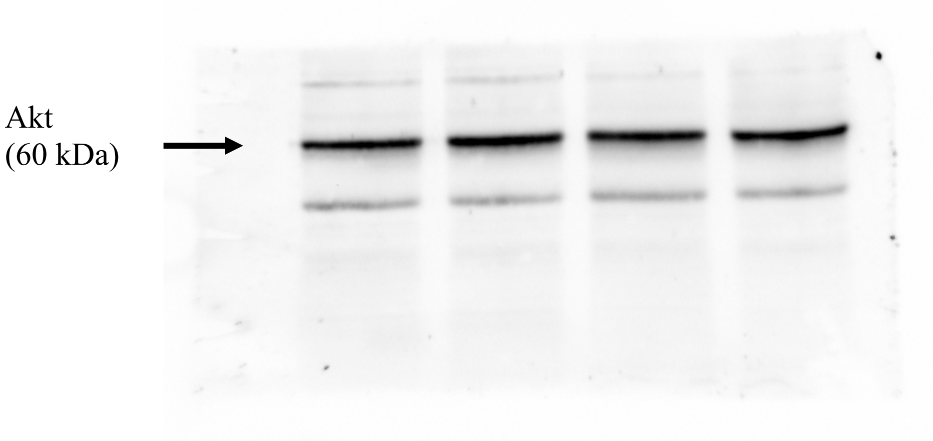

P-GSK-3β in NSCs cells (Figure 2I)

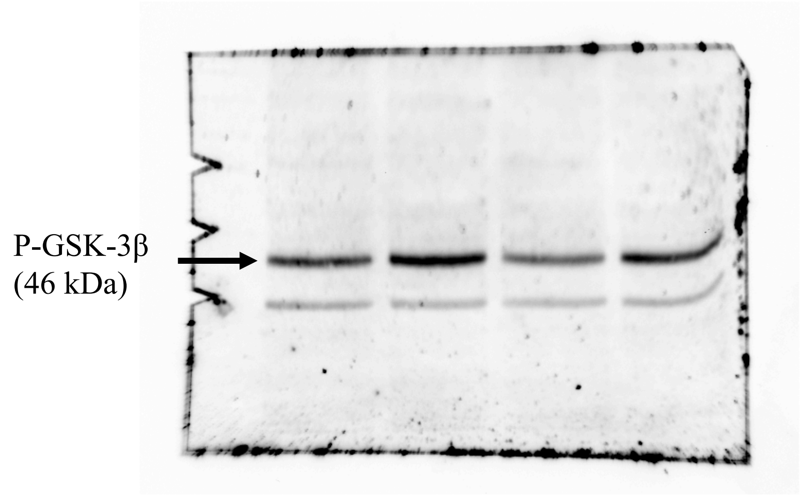

GSK-3β in NSCs cells (Figure 2I)

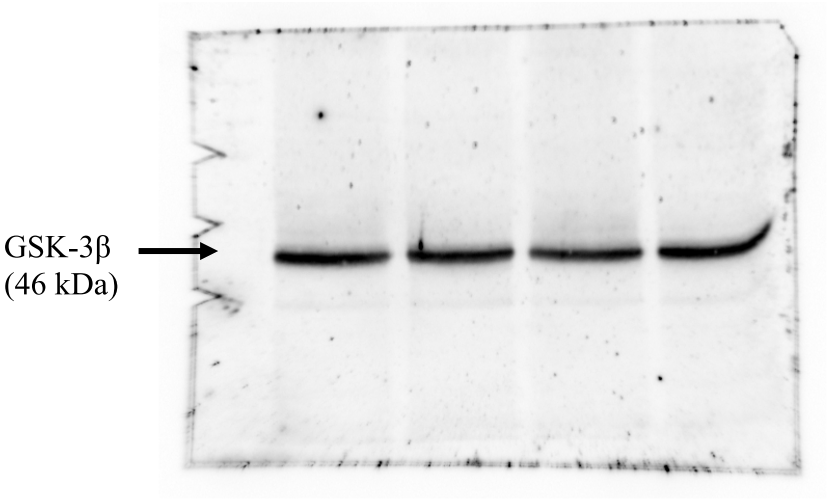

P-Tau in NSCs cells (Figure 2J)

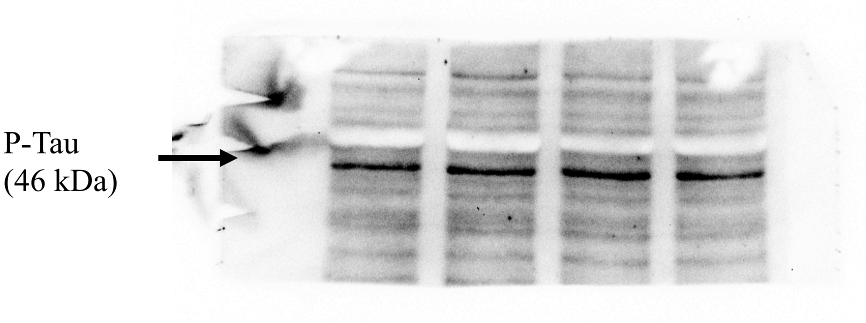

Tau in NSCs cells (Figure 2J)

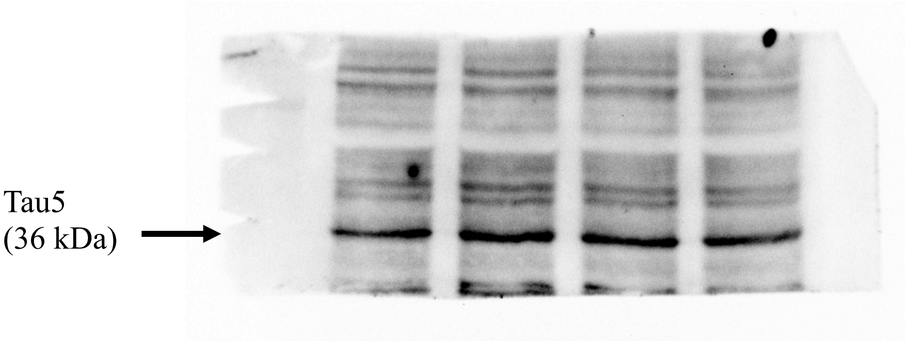

Actin for Tau in NSCs cells (Figure 2J)

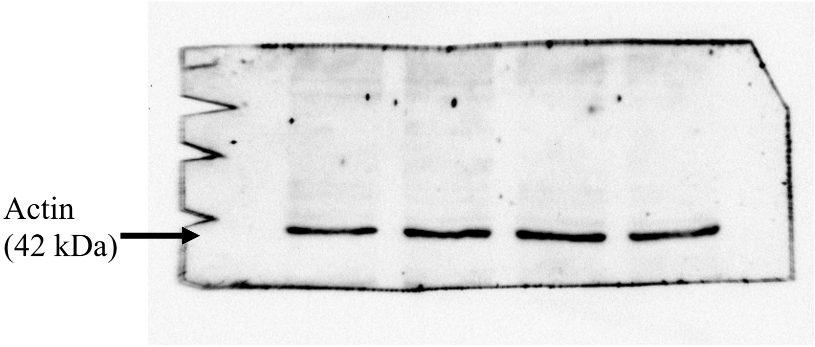

P-p65 in NSCs cells (Figure 2L)

p-P65
(65 kDa)

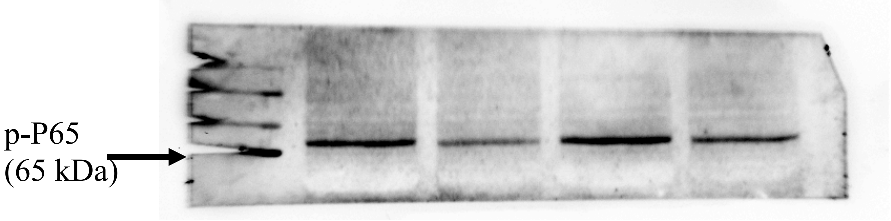

p65 in NSCs cells (Figure 2L)

p65
(65 kDa)

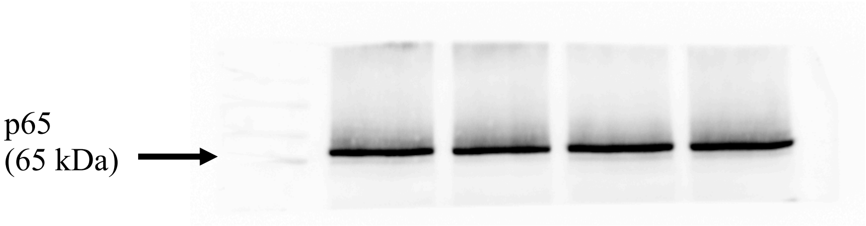

GHRH-R and SV1 expression in SH-SY5Y cells (Figure 3A)

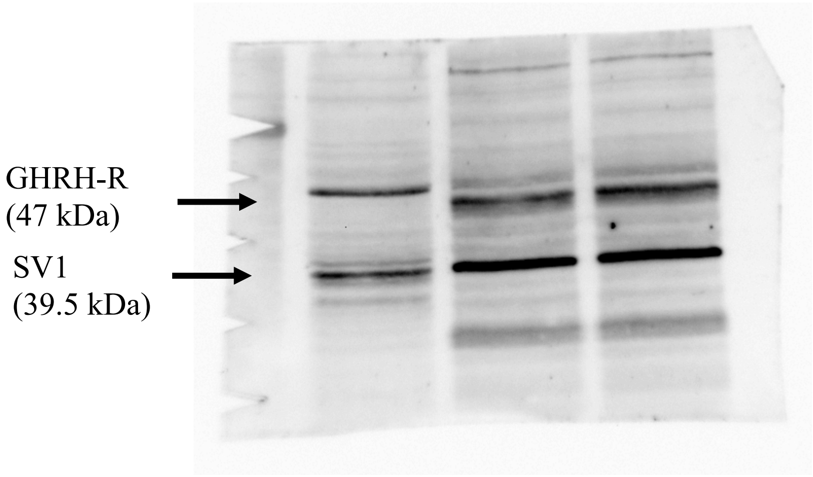

Actin for GHRH-R and SV1 expression in SH-SY5Y cells (Figure 3A)

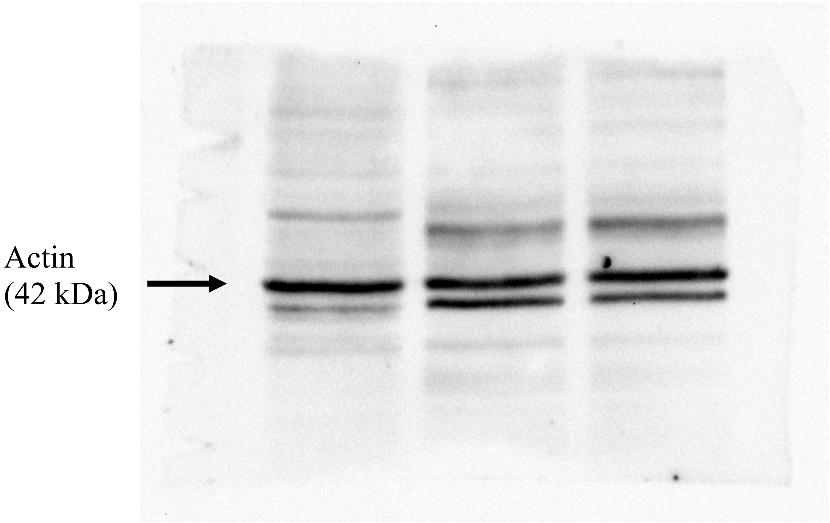

GHRH expression in SH-SY5Y cells (Figure 3B)

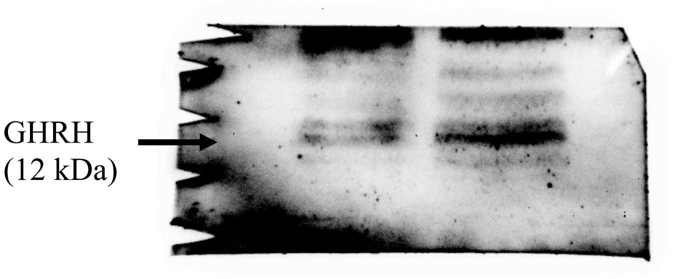

Actin for GHRH expression in SH-SY5Y cells (Figure 3B)

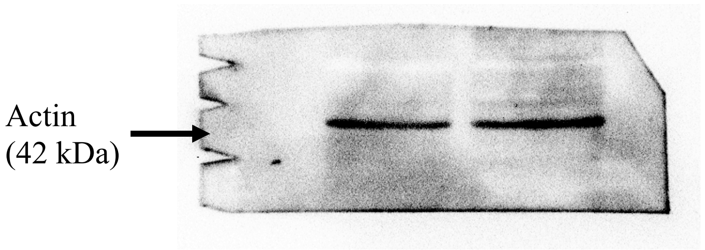

NeuN in SH-SY5Y cells (Figure 3E)

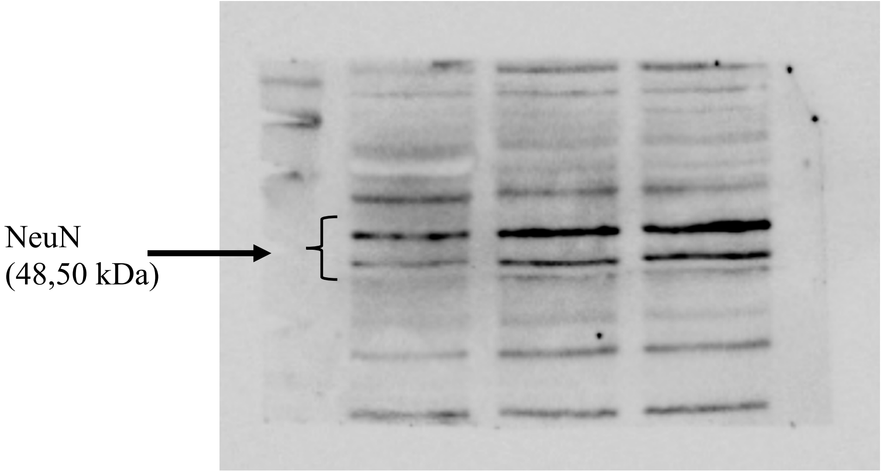

Actin for NeuN in SH-SY5Y cells (Figure 3E)

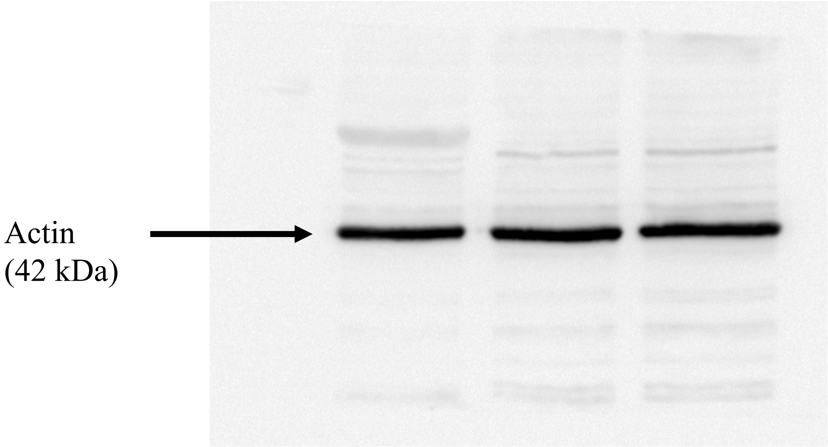

P-GSK-3β in SH-SY5Y cells (Figure 3J)

P-GSK-3β
(46 kDa)

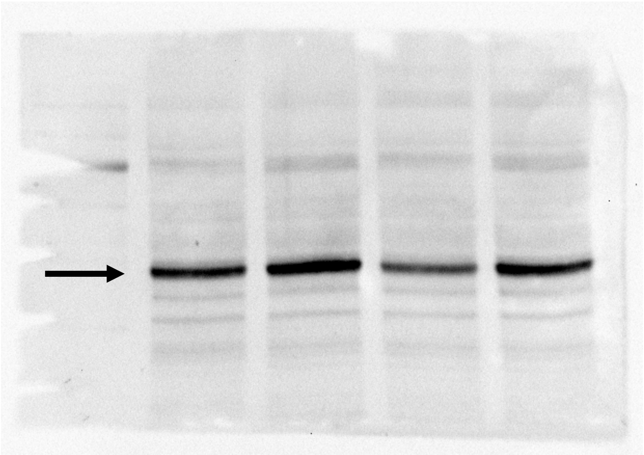

GSK-3β in SH-SY5Y cells (Figure 3J)

GSK-3β
(46 kDa)

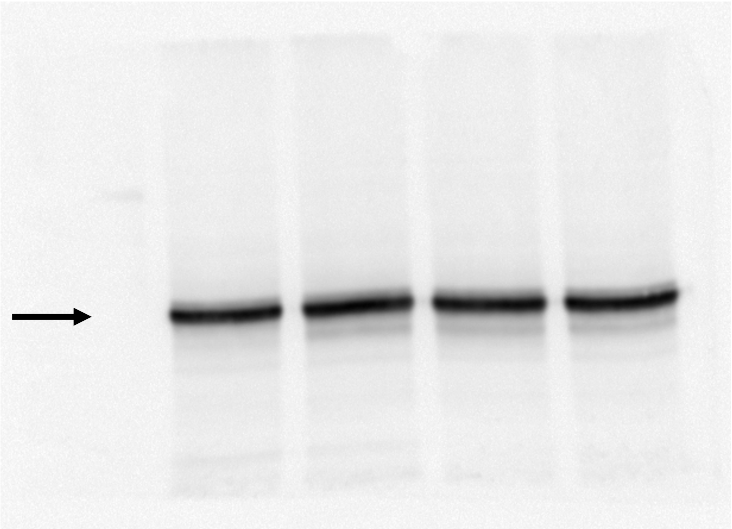

P-Tau in SH-SY5Y cells (Figure 3K)

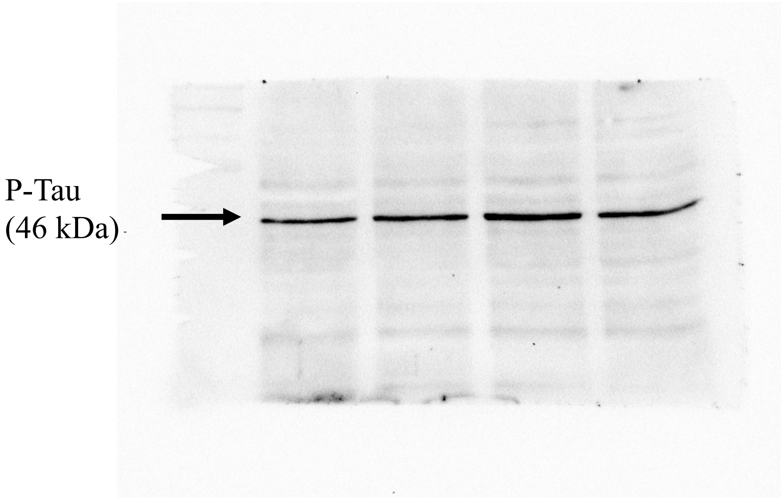

Tau in SH-SY5Y cells (Figure 3K)

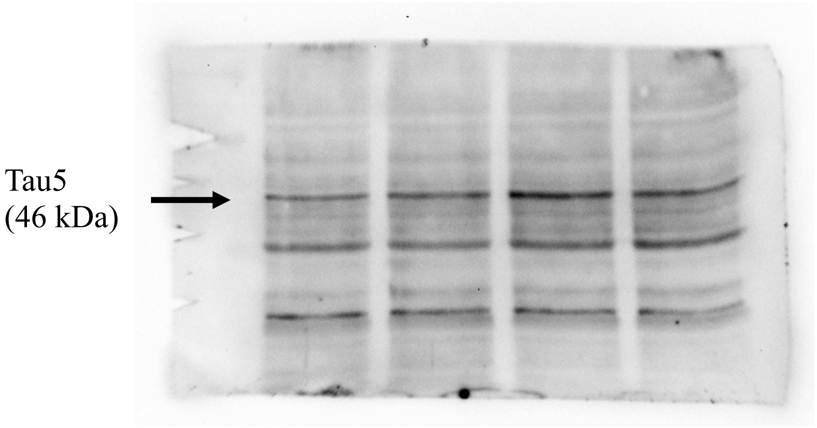

BACE1 in 5XFAD mouse brain lysates (Figure 4H)

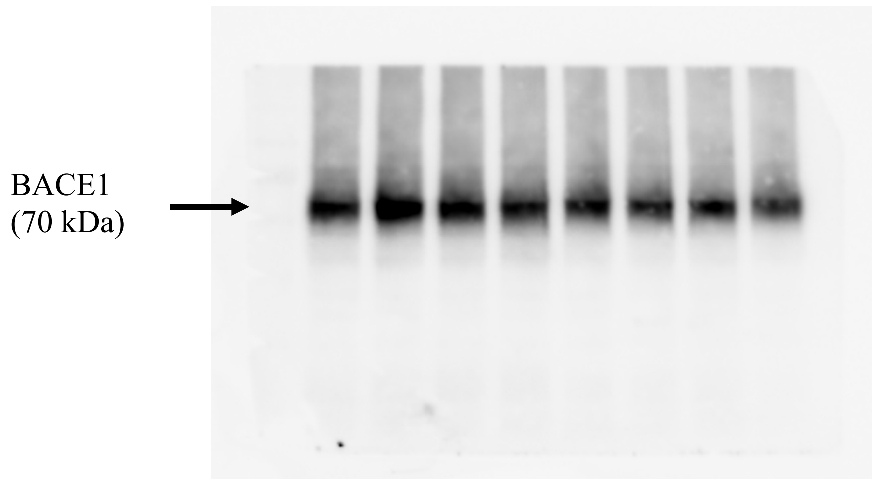

Actin for BACE1 in 5XFAD mouse brain lysates (Figure 4H)

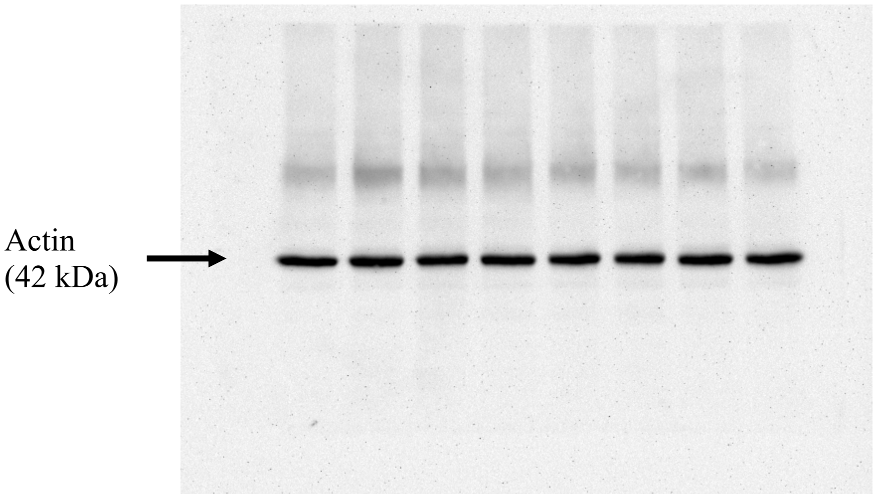

P-GSK-3β in 5XFAD mouse brain lysates (Figure 4I)

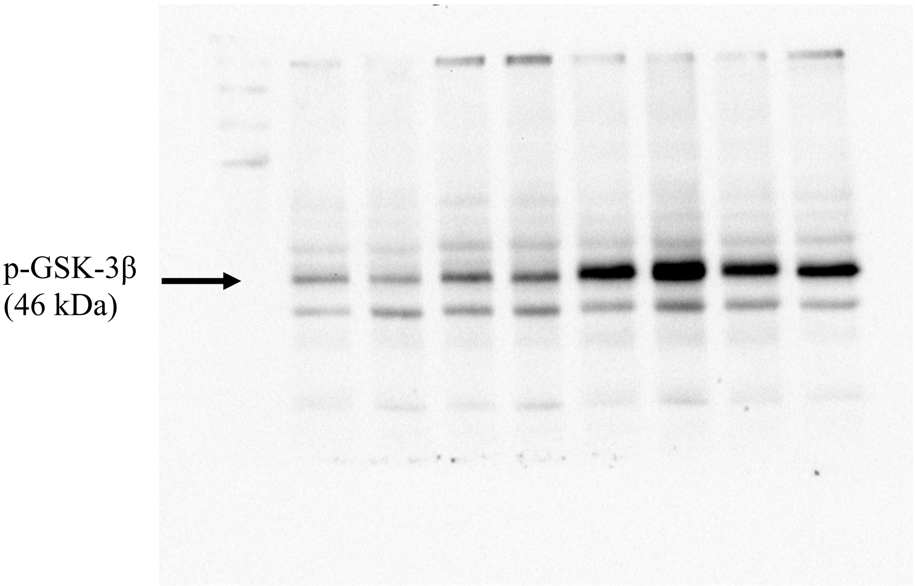

GSK-3β in 5XFAD mouse brain lysates (Figure 4I)

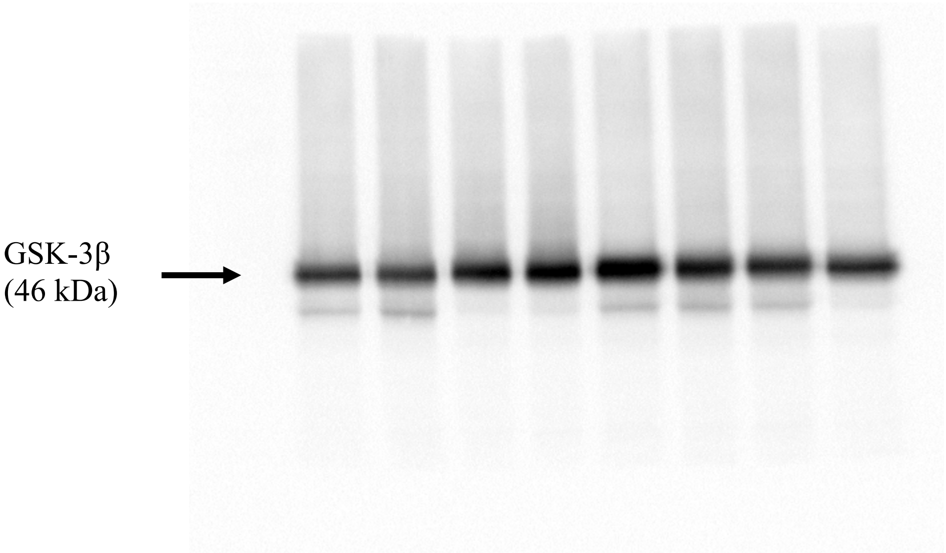

CDK5 in 5XFAD mouse brain lysates (Figure 4J)

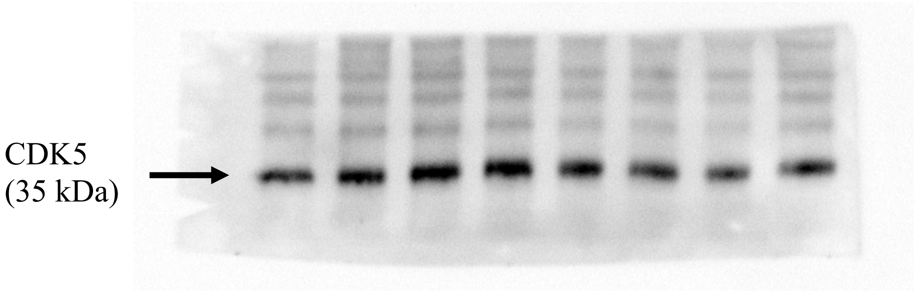

Actin for CDK5 in 5XFAD mouse brain lysates (Figure 4J)

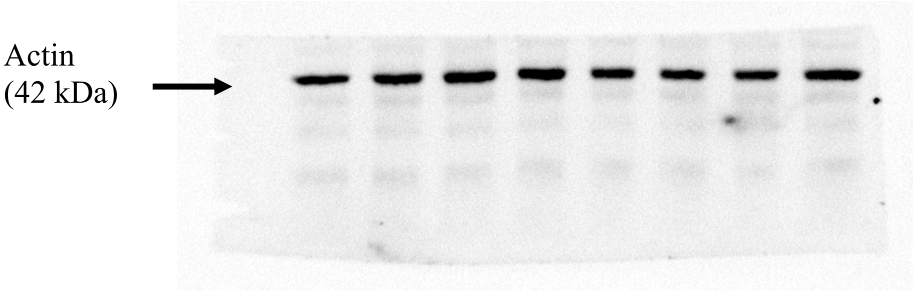

P-Tau in 5XFAD mouse brain lysates (Figure 4K)

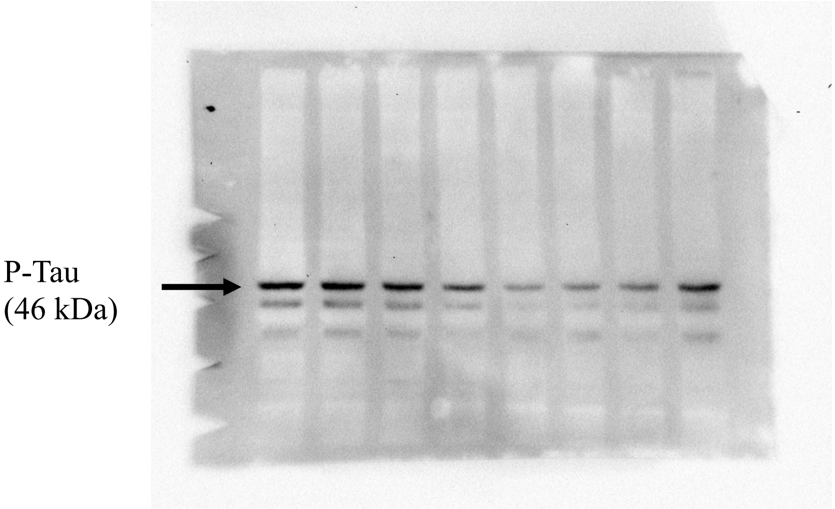

Tau in 5XFAD mouse brain lysates (Figure 4K)

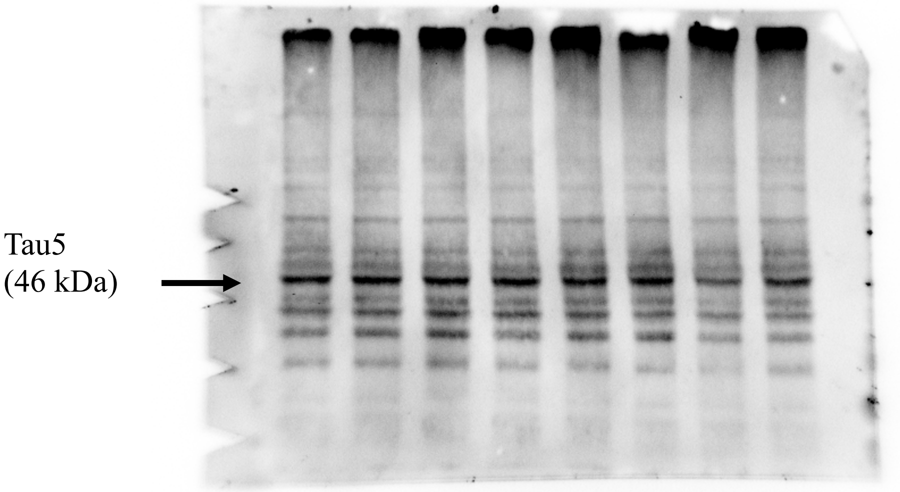

P-p65 in mouse brain lysates (Figure 5H)

p-P65
(65 kDa)

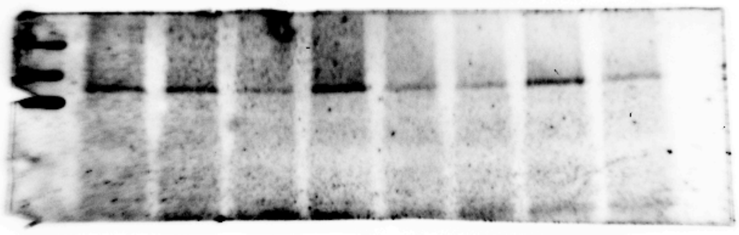

p65 in mouse brain lysates (Figure 5H)

p65
(65 kDa)

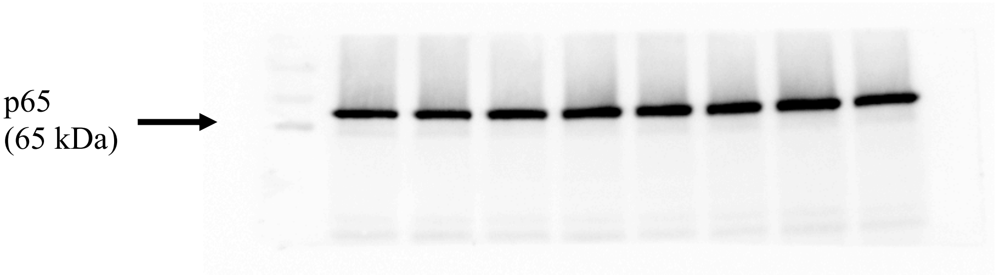

NeuN in mouse brain lysates (Figure 6A)

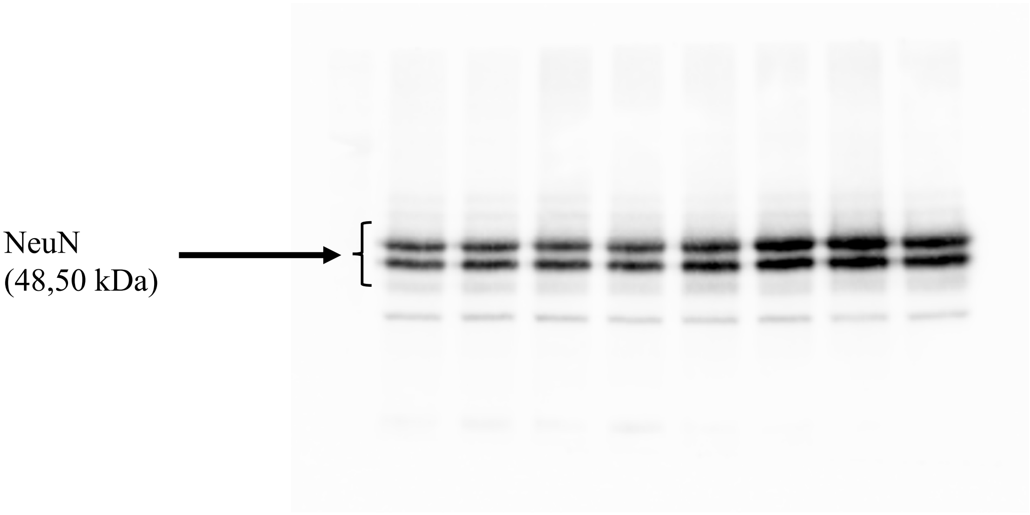

Actin for NeuN in mouse brain lysates (Figure 6A)

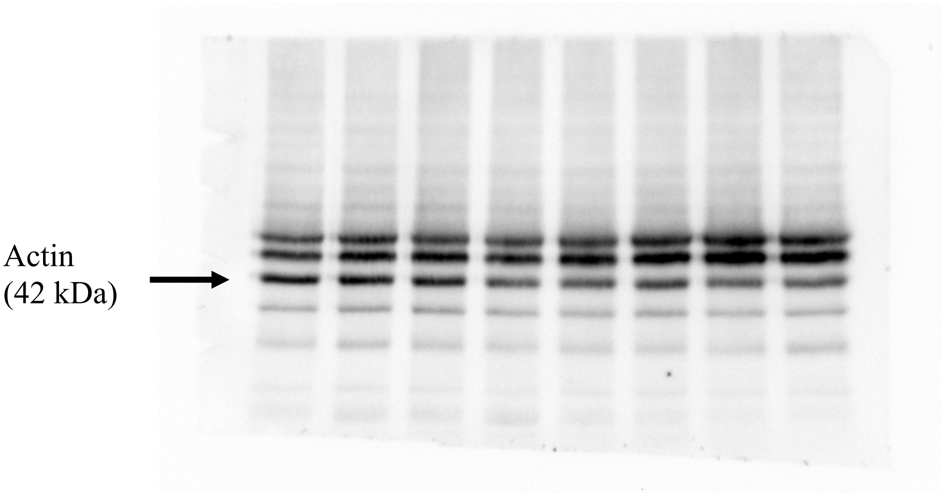

Synaptophysin in mouse brain lysates (Figure 6B)

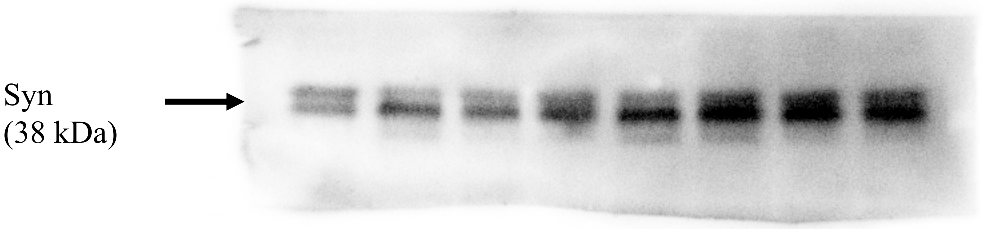

Actin for Synaptophysin in mouse brain lysates (Figure 6B)

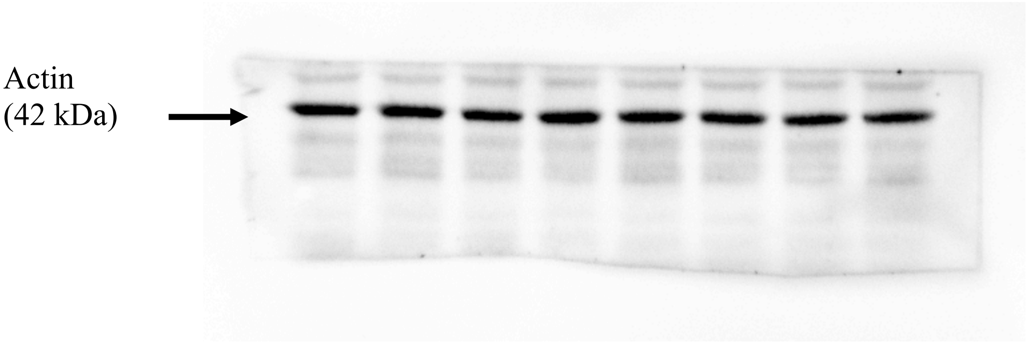

P-Akt in mouse brain lysates (Figure 6D)

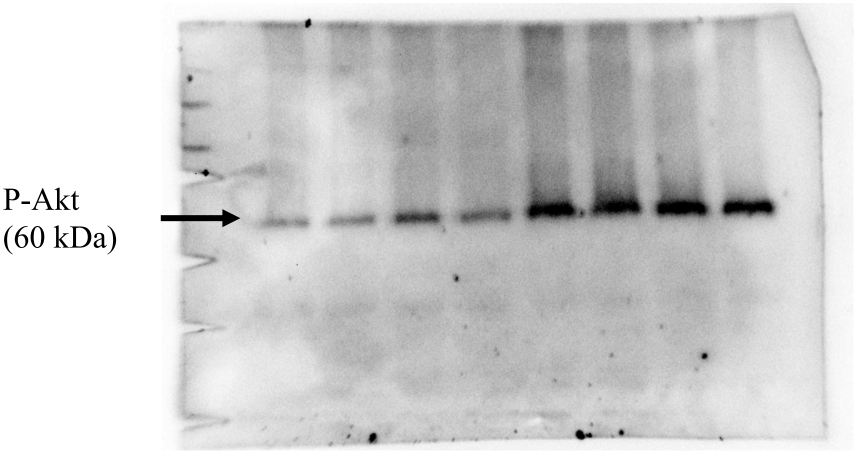

Akt in mouse brain lysates (Figure 6D)

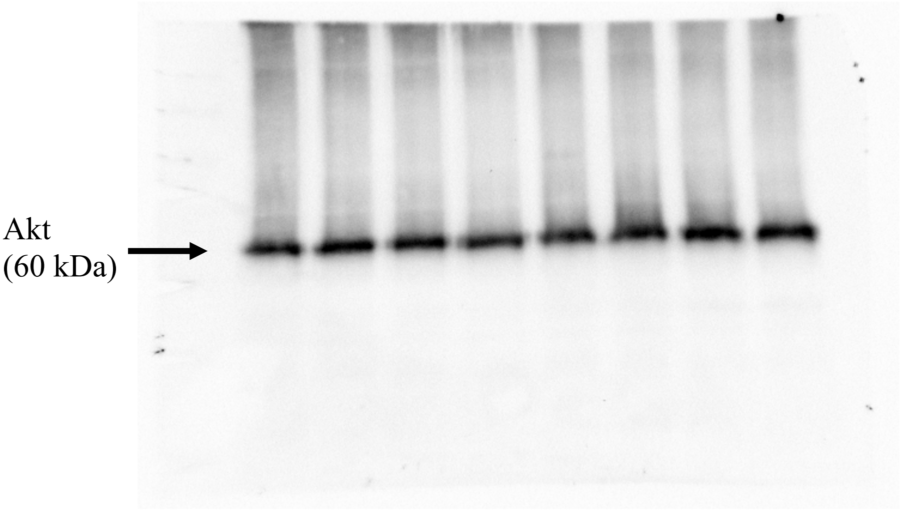

P-CREB in mouse brain lysates (Figure 6E)

P-CREB
(43 kDa)

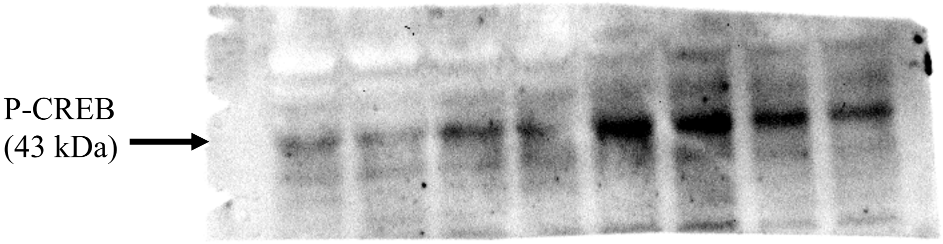

CREB in mouse brain lysates (Figure 6E)

CREB
(43 kDa)

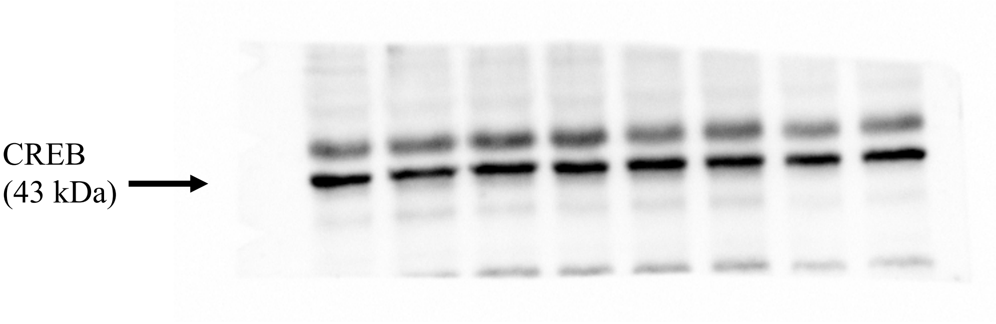

Supplement: Supplementary file 2 — Original Western blots [file 41419_2026_8699_MOESM2_ESM.pdf]
